# Single-cell multi-omic atlas and morphogen screening informs midbrain and hindbrain organoid engineering

**DOI:** 10.1038/s41593-026-02316-x

**Published:** 2026-06-03

**Authors:** Nadezhda Azbukina, Zhisong He, Hsiu-Chuan Lin, Malgorzata Santel, Bijan Kashanian, Ashley Maynard, Tivadar Török, Ryoko Okamoto, Marina T. Nikolova, Makiko Seimiya, Sabina Kanton, Valentin Brösamle, Rene Holtackers, J. Gray Camp, Barbara Treutlein

**Affiliations:** 1 Department of Biosystems Science and Engineering, ETH Zürich, Basel, Switzerland; 2 Max Planck Institute for Evolutionary Anthropology, Leipzig, Germany; 3 Institute of Human Biology (IHB), Roche Pharma Research and Early Development, Roche Innovation Center Basel, Basel, Switzerland; 4 Biozentrum, University of Basel, Basel, Switzerland

**Keywords:** Gene regulatory networks, Multicellular systems, Developmental neurogenesis, Functional genomics

## Abstract

Patterning of the neural tube establishes midbrain and hindbrain structures that coordinate motor movement, process sensory input and integrate cognitive functions. Cellular impairment within these structures underlies diverse neurological disorders, and in vitro organoid models promise inroads to understanding development and modeling disease. Here, we use paired single-cell transcriptome and accessible chromatin sequencing to map cell composition and regulatory mechanisms in organoid models of midbrain and hindbrain. We find that existing midbrain organoid protocols generate ventral and dorsal cell types, covering regions including floor plate, dorsal and ventral midbrain and adjacent hindbrain regions. Gene regulatory network inference and transcription factor perturbation resolve mechanisms underlying neuronal differentiation. A single-cell multiplexed patterning screen identifies morphogen concentrations that expand existing organoid models, including conditions generating medulla glycinergic neurons and cerebellum glutamatergic subtypes. Together, the multi-omic atlas and morphogen screen reveal morphogen–regulon relationships guiding region-specific progenitor differentiation towards diverse neuron types of the posterior brain.

## Abstract (web summary)

By combining single-cell sequencing and morphogen screening, researchers map how signaling cues shape cell identity in mid-hindbrain organoids and identify conditions that generate novel posterior brain neuron types.

## Main

The midbrain and hindbrain function to coordinate motor movements and process sensory inputs^1^, and their impairment is associated with diverse disorders^2^, including brain malformations and Parkinson’s disease. During development, the mesencephalon (midbrain) and rhombencephalon (hindbrain) are two of the three primary brain vesicles emerging from the anterior part of the neural tube. These regions develop into the cerebral peduncles, tegmentum and tectum (midbrain) and the medulla, pons and cerebellum (hindbrain) of the human brain. The midbrain–hindbrain boundary serves as a critical organizing center, where fibroblast growth factor 8 (FGF8) and WNT morphogen signaling establish the isthmic organizer that patterns both regions through reciprocal signaling gradients^3^. A transcriptional boundary between OTX2 (midbrain) and GBX2 (hindbrain) expression domains is maintained through cross-repression, defining the fundamental antero-posterior (AP) axis of the posterior brain^4^. Midbrain specification relies on the FGF8–WNT1 signaling hub at the isthmus, which induces a series of transcriptional regulatory events that coordinate progenitor proliferation, identity and differentiation, including factors such as EN1/2, NGN2 and MASH1 (ASCL1)^5,6^. Importantly, this regulatory network directs the specification of midbrain dopaminergic neurons through downstream activation of LMX1a, MSX1 and NURR1 (ref. ^7^). By contrast, hindbrain development involves rhombomere segmentation, each specified by distinct HOX gene expression patterns working in concert with cofactors MEIS and PBX^8^. Retinoic acid (RA) gradients provide essential AP patterning cues to create rhombomeric boundaries^9^. Leveraging these developmental principles can provide an opportunity to generate posterior brain cells and tissues in vitro.

Human brain (or neural) organoids are three-dimensional models generated from human embryonic stem cells or induced pluripotent stem cells (iPS cells) that recapitulate certain aspects of brain development. Brain organoids provide an accessible gateway to obtain diverse human neural phenotypes and recapitulate intercellular connections and interactions. Protocols have been established to guide organoid differentiation into broad and specific brain regions, providing functional models to understand the mechanisms underlying human brain development^10^ and neurodevelopmental disorders^11^. Insights from developmental biology, including timed manipulation of Sonic Hedgehog (SHH), WNT, FGF, bone morphogenetic protein (BMP) and RA signaling pathways, have been leveraged to generate midbrain^12–15^, hindbrain^16,17^, cerebellum^18,19^ and brainstem organoids^20,21^. Single-cell transcriptomic profiling (scRNA-seq) has illuminated brain organoid cell type diversity, and comparisons to primary developing neural tissue counterparts have helped assess organoid fidelity. A recent effort to integrate existing scRNA-seq data from neural organoids revealed an underrepresented coverage of posterior brain region neuron populations in existing organoid sequencing data^22^. It remains unclear how to generate certain specific regions of the posterior brain, and how to effectively control the co-development of multiple regions of interest within the same neural tissue^23^.

Organoids provide scalable inroads to screen and optimize morphogen timing, concentration and combinations^24–27^. Recent efforts have investigated midbrain–hindbrain fate induction, typically through modulation of WNT and SHH signaling^25^ or by proposing a specific regimen of growth factors^19,26^; however, a systematic manipulation of posterior brain patterning is lacking. Therefore, it is unclear how morphogen combinations converge on transcriptional regulation to robustly and reproducibly guide midbrain-like and hindbrain-like cell-type specification within complex human neural tissues. In this study, we first generated a single-nucleus multi-omic atlas profiling transcriptome and accessible chromatin from the same single cells over a time course of posterior brain organoid development. We assessed how in vitro models capture not only the transcriptomic, but also the regulatory landscape of these complex brain regions. We inferred cell-type-specific transcription factor (TF) regulatory networks and used single-cell CRISPR perturbation experiments to dissect cell state and regional fate establishment. Finally, we conducted a multiplexed morphogen patterning screen with single-cell transcriptomic readout of individual organoids in replicates, testing 48 concentrations and combinations of ten morphogens involved in brain patterning. We identified conditions driving the emergence of underrepresented cell types, such as medulla glycinergic neurons and cerebellum glutamatergic neurons, thereby expanding existing mesencephalon and rhombencephalon organoid models. Together, our findings present a single-cell multi-omic (scMultiome) atlas and morphogen screen of human posterior brain organoid development, advancing our understanding of its complexity and developmental dynamics.

## Results

### Multi-omic cell atlas of posterior brain organoids identifies features recapitulating primary brain development

**Fig. 1:**
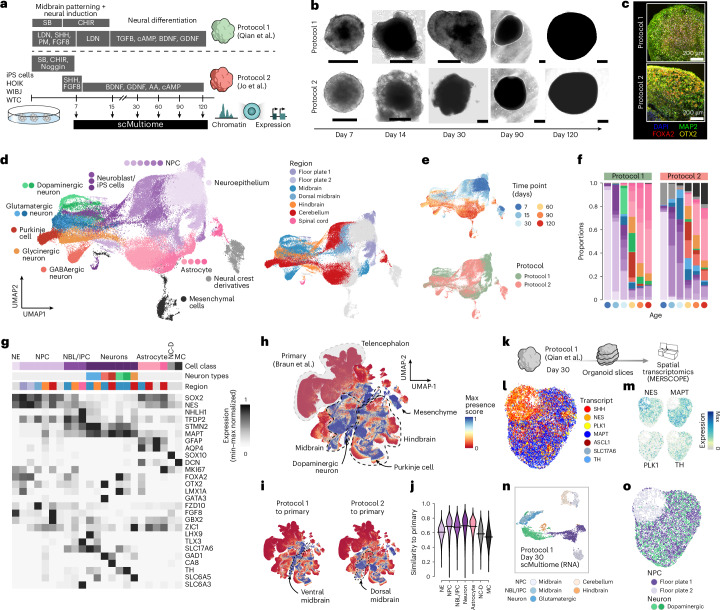
Single-cell atlas of human posterior neural organoid development. **a**, Schematic of the two protocols generating midbrain/posterior brain organoids and the single-cell multi-omic measurements over a time course. **b**, Bright-field images of organoids showing examples of different stages of organoid development. Scale bar is 500 µm. **c**, Immunofluorescence staining of MAP2, FOXA2 and OTX2 on organoids generated using the two protocols. **d**,**e**, UMAP of the posterior neural organoid developmental cell atlas based on gene expression profiles, colored by the annotation of cell types (left) and regions (right) (**d**), as well as experimental meta information, including time point (top) and protocol (bottom) (**e**). IPC, intermediate progenitor cell. **f**, Temporal profiles of cell type proportions in organoids generated using the two protocols. **g**, Heatmap of gene expression levels of selected cell type markers (row) across different cell types (columns). **h**,**i**, UMAP of the human developing brain cell atlas colored by cell presence within the posterior neural organoid atlas (max presence scores), for both protocols together (**h**) or each of the two protocols separately (**i**). **j**, Distributions of transcriptomic similarity between major cell classes in the posterior neural organoid atlas and their primary counterparts. NE, neuroepithelium; NBL, neuroblast; NC-D, neural crest derivative; MC, mesenchymal cell. **k**, Schematic of spatial transcriptomic measurement on organoids based on protocol 1 at day 30 with MERSCOPE. **l**, Spatial distribution of selected transcript species in an example organoid section. **m**, Expression levels of *NES*, *MAPT*, *PLK1* and *TH* in the segmented cells of the example organoid section. **n**, UMAP of a subset of the posterior neural organoid atlas, with the matched protocol and time point corresponding to the MERSCOPE data, colored by annotated cell types. **o**, Transferred cell type labels of the segmented cells in the example organoid section.

**Extended Data Fig. 1:**
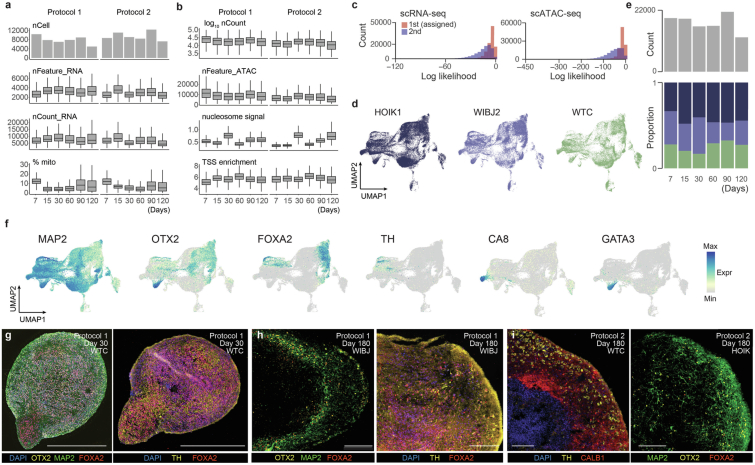
Quality control analysis of time-course single-cell multi-omic data. (**a, b**) Quality control of the time-course snRNA-seq (**a**) and snATAC-seq (**b**) in 2 protocols. Each column corresponds to one time point. nCell, number of cells; nCount_RNA, total number of RNA molecules detected within a cell; nFeature_RNA, total number of genes detected within a cell; %mito, percentage of mitochondrial genes; nCount, number of detected reads; nFeature_ATAC, number of peak region fragments; TSS, transcription start site. Exact sample sizes for panel **a**) and **b**) could be found in Supplementary Table 1. (**c**) Histograms showing assignment log-likelihoods for iPSC line demultiplexing based on single nucleotide variants. (**d**) Distribution of iPSC lines on the UMAP embedding. (**e**) Bar plot showing the number of cells (top) and stacked barplot showing the proportion of cell lines (bottom) across different time points. (**f**) UMAP embedding colored by marker gene expression (log-transformed transcript counts per 10k + 1). (**g**) Immunofluorescence staining of MAP2, FOXA2, TH and OTX2 in organoids generated with protocol 1, WTC line, day 30. Scale bar corresponds to 500um. (**h**) Immunofluorescence staining of MAP2, FOXA2, TH and OTX2 in organoids generated with protocol 1, day 180. Scale bar corresponds to 200um. (**i**) Immunofluorescence staining of MAP2, FOXA2, OTX2, TH and CALB1 in organoids generated with protocol 2, day 180. Scale bar corresponds to 200um.

**Extended Data Fig. 2:**
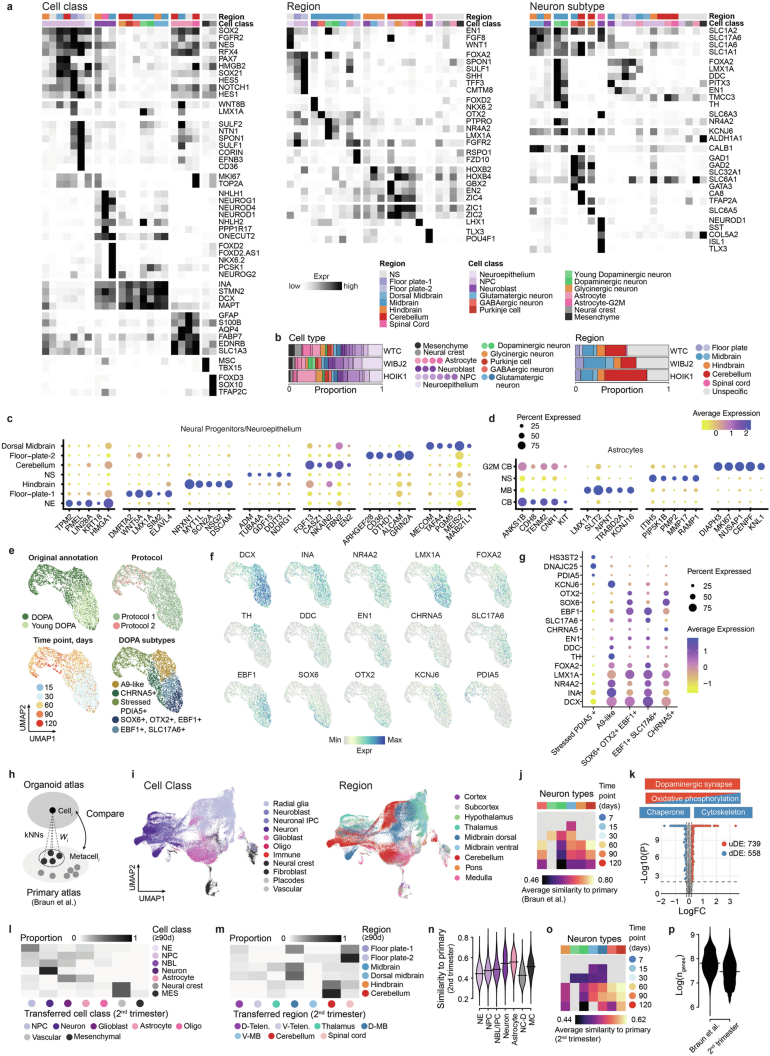
Supplemental analysis of time-course single-cell transcriptomic data. (**a**) Heatmaps showing min-max scaled mean expression of marker genes for each cell type. Genes are grouped by cell class markers (left), region markers (center), and neuronal subtypes markers (right). (**b**) Stacked barplots showing proportions of cell types (left) and brain regions (right) across different iPSC lines. (**c,d**) Dotplot showing expression of novel marker genes for Neural Progenitors/Neuroepithelium clusters (**c**) and Astrocytes (**d**). CB, cerebellum; NS, non-specific; MB, midbrain. (**e**) UMAP embedding of the dopaminergic neurons subset, colored by original cell type annotation, protocol, time point, dopaminergic neurons subtypes. (**f**) UMAP embedding of dopaminergic neurons, colored by marker gene expression (log-transformed transcript counts per 10k + 1). (**g**) Dotplot showing marker gene expression in dopaminergic neurons subtypes. (**h**) Schematic of atlas projection to the primary human developing brain scRNA-seq cell atlas. (**i**) UMAP embedding colored by the scArches-transferred cell class (left) and region (right) labels from the primary reference. (**j**) Heatmap showing average similarity of neuronal subtypes to their primary counterparts. (**k**) Volcano plot showing differentially expressed genes (F-test, one-sided, p-value adjusted with Bonferroni method, details see Methods) between dopaminergic neurons in the brain organoid atlas and primary dopaminergic neurons. (**l-m**) Heatmap of the proportions of transferred cell class (**l**) or regional (**m**) labels for each annotated cell class in the d90 and d120 samples. (**n**) Distributions of transcriptomic similarity between major cell classes in the posterior neural organoid atlas and their primary counterparts in the 2nd-trimester atlas. (**o**) Heatmap showing average similarity of neuronal subtypes to primary counterparts in the 2nd-trimester atlas. (**p**) Distribution of log-transformed detected gene number per cell in the 1st-trimester developing human brain atlas and the integrated 2nd-trimester atlas (2nd-Trimester).

We generated a time-course single-nucleus multi-omic dataset with paired measurements of transcriptomes and accessible chromatin to map cellular composition and gene regulatory dynamics in two previously established protocols modeling midbrain development^14,15^ (Fig. 1, Extended Data Fig. 1 and Supplementary Table 1). These protocols use the WNT pathway activator CHIR-99021 (CHIR), SHH and FGF8 to pattern the neural epithelium in a continuous (protocol 1) or sequential (protocol 2) manner, respectively (Fig. 1a–c). The dataset incorporates 104,452 cells collected from five time points between day 7 and day 120 of organoid development from three human iPS cell lines, covering periods of regionalization, neurogenesis and maturation. We observed time-dependent trajectories from early neuroepithelial cells via neural progenitor cells (NPCs) to multiple neuronal cell populations, including dopaminergic, glutamatergic, GABAergic and glycinergic neurons (Fig. 1d–g, Extended Data Fig. 1a–i, Extended Data Fig. 2a–d and Supplementary Table 2). Interestingly, both protocols generated a substantial proportion (57%) of neurons with hindbrain rather than midbrain signatures, including glycinergic neurons and Purkinje cells, suggesting successful posteriorization without restriction to midbrain cell types. Notably, the two protocols generated distinct neuronal populations (Fig. 1e–g). Protocol 1 produced the majority (89%) of dopaminergic neurons, with multiple subtypes including *KCNJ6*^+^ A9-like dopaminergic neurons and *SOX6*^+^*OTX2*^+^ populations (Extended Data Fig. 2e–g). Protocol 2 produced the majority (82%) of glutamatergic neurons as well as of midbrain GABAergic neurons (91% of GABAergic neurons). In both protocols, a shift from neurogenesis to gliogenesis was observed after 2 months of organoid cultures (Fig. 1f,g). We did not observe dramatic differences in cell type and state distribution between different cell lines (Extended Data Figs. 1e and 2b).

We next compared organoid data with a human first-trimester developing brain cell atlas^28^ using transfer learning. Projection-based cell matching, as indicated by the maximum presence score per cell in the primary atlas (Fig. 1h,i) and transferred cell class and regional labels (Extended Data Fig. 2h,i), confirmed that cells produced with the two protocols matched to primary counterparts. Protocol 1 mapped to ventral midbrain fates, while protocol 2 mapped to dorsal midbrain identities. Transcriptomic comparison between NPCs, neuroblasts, intermediate progenitor cells and neurons in organoids showed high transcriptomic similarity to matched primary metacells (Fig. 1j). Interestingly, there were time-dependent patterns of transcriptomic similarity to primary neurons, with most neuron cell types peaking in similarity at 1–2 months of culture (Extended Data Fig. 2j). Differential expression analysis suggests that metabolism and synaptic programs may drive the divergence between neurons in organoids and those in the human developing brain (Extended Data Fig. 2k and Supplementary Table 3). Comparison to the second-trimester developing human brain atlas showed consistent cell class and regional label mapping results (Extended Data Fig. 2l–p).

**Extended Data Fig. 3:**
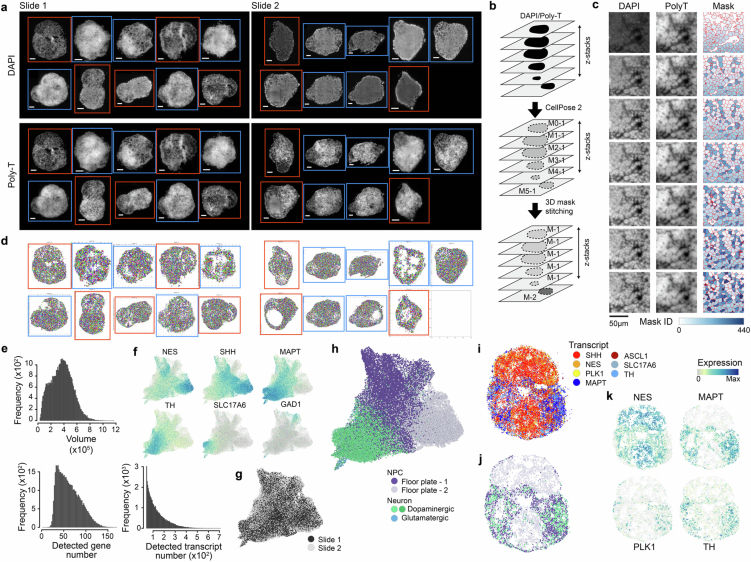
Supplementary analysis of the spatial transcriptomics data. (**a**) DAPI and polyT signal for each slide. Orange and blue frames indicate tissue sections from the two different organoids. The experiment was performed using 2 different organoids. Scale bars: 100µm. (**b**) Schematic illustrating the MERFISH data analysis pipeline. The labeled polygons represent segmented cell masks. (**c**) Example images, showing a cropped section from a spatial transcriptomic slide. The left column shows the DAPI signal, the middle shows the polyT signal, and the right shows the inferred cell masks. (**d**) Slices, showing MERFISH segmented nucleus masks. (**e**) Histograms showing distribution of segmented cell volumes (top), detected genes per cell (bottom left), and detected transcripts per cell (bottom right). (**f**) UMAP embedding of segmented cells, based on their summarized transcriptomic features, colored by marker gene expression. (**g**) UMAP embedding colored by slide. (**h**) UMAP embedding colored by cell type labels, transferred from corresponding scRNA-seq data. (**i-k**) Example of organoid slice profiled on slide 2, showing selected transcript detection (**i**), transferred cell type label (**j**), marker gene expression (log-transformed transcript counts per 10k + 1) (**k**).

We applied multiplexed error-robust fluorescence in situ hybridization (MERFISH, adapted by Vizgen MERSCOPE) to characterize tissue architecture of day 30 organoids (protocol 1) (Fig. 1k). We observed a patterned organization of two distinct regions demarcated by the expression of either *SHH* and *NES* or *MAPT* and *TH* (Fig. 1l,m and Extended Data Fig. 3a–e). We annotated segmented cells through correlation-based label transfer using protocol and time-point matched counterparts from the multi-omic cell atlas (Fig. 1n,o and Extended Data Fig. 3f–h). This analysis revealed distinct spatial cell type compositions, where *SHH*^+^/*NES*^+^ regions were enriched for NPC floor plate type-2 cells, and *MAPT*^+^/*TH*^+^ regions were enriched for NPC floor plate type-1 and dopaminergic neurons (Extended Data Fig. 3i–k). Notably, NPC floor plate type-1 cells are transcriptomically more similar to dopaminergic neurons compared to the other floor plate NPCs, which implies the coordination of spatial patterning and neural differentiation.

### Chromatin accessibility dynamics during human posterior brain organoid development

**Fig. 2:**
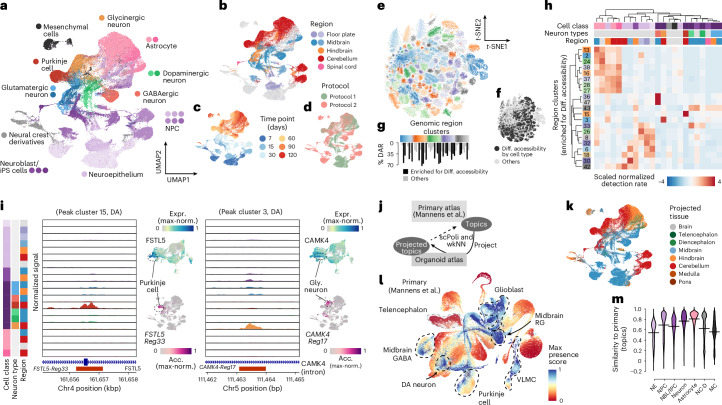
Chromatin accessibility dynamics during human posterior brain organoid development. **a**–**d** UMAP of the human posterior brain organoid cell atlas, based on chromatin accessibility profiles, colored by annotated cell type (**a**), regional identity (**b**), time point (**c**) and protocol (**d**). **e**,**f**, *t*-distributed stochastic neighbor embedding (*t*-SNE) of chromatin accessible regions, colored by the identified anatomical region clusters (**e**) and whether regions are differentially accessible in specific cell types (**f**). **g**, Proportions of differentially accessible regions (DARs) in region clusters. **h**, Heatmap showing average relative accessibility patterns across cell types (columns) for different region clusters (rows). **i**, Chromatin accessibility profile tracks in different cell types at two representative loci: *FSTL5* and *CAMK4*. DA, differentially accesible. **j**, Schematic of topic-based projection to the primary human developing brain scATAC-seq cell atlas. **k**, UMAP of the human posterior brain organoid cell atlas, colored by transferred tissue labels from the primary cell atlas. **l**, UMAP of the human developing brain cell atlas colored by cell presence within the posterior neural organoid atlas (max presence scores). DA, dopaminergic. **m**, Distribution of chromatin accessibility topic profile similarity between major cell classes in the posterior neural organoid atlas and their primary counterparts.

**Extended Data Fig. 4:**
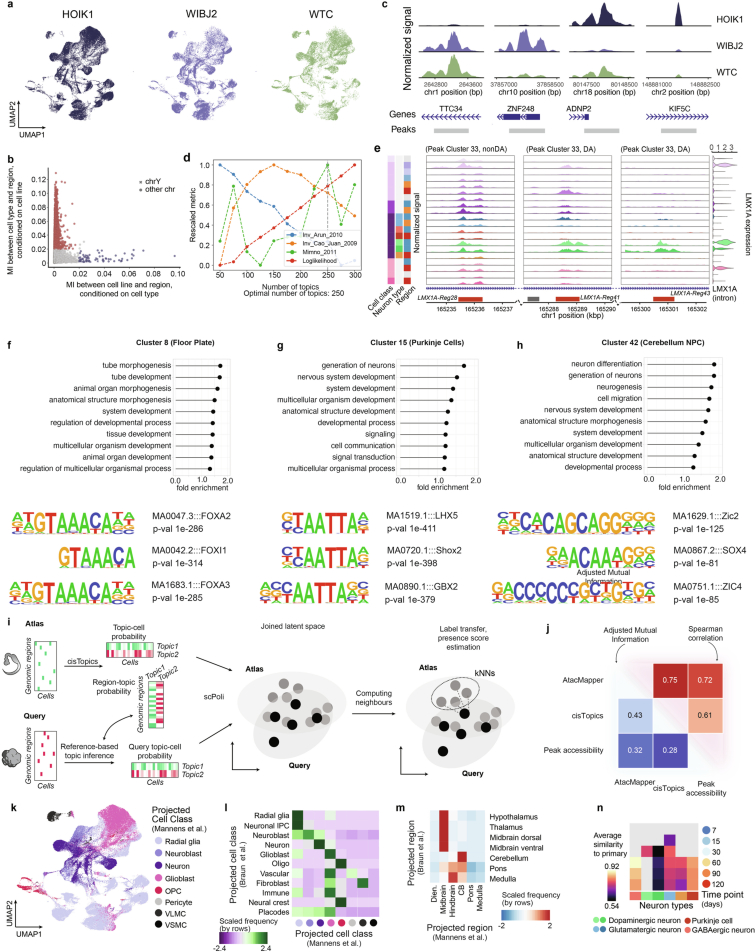
Supplemental analysis of time course single-cell chromatin accessibility data. (**a**) Distribution of iPSC lines on the UMAP embedding of single-cell chromatin accessibility data. (**b**) Scatterplot showing mutual information between cell line and open chromatin region, and cell type and open chromatin region for each peak. The shape of each point indicates whether the peak is mapped to the Y chromosome. (**c**) Chromatin accessibility profile tracks in different cell types at 4 representative loci, shown to be cell line-specific. (**d**) Visualization of metric values for models with different numbers of topics. All metrics are min-max scaled, Arun and Cao metrics were inverted for visualization purposes. (**e**) Chromatin accessibility profile tracks in different cell types at three representative loci for *LMX1A* gene. Violin plots on the right show *LMX1A* expression split by cell type. (**f-h**) Cluster characterisation. Examples of enriched GO-biological process terms from GREAT analysis of DA peaks of the indicated cluster (top) and enriched motifs (bottom) for cluster 8 (**f**), 15 (**g**) and 42 (**h**). All adjusted p-values of presented terms were < 10^−30, one-sided hypergeometric test over genes. Fold enrichment is also derived from hypergeometric test over genes. (**i**) Schematic illustrating the AtacMapper framework for mapping query scATAC-seq data to reference atlases. (**j**) Heatmap showing the adjusted mutual information between transferred labels (bottom triangle, blue), and Spearman correlation (top triangle, red) using AtacMapper, cisTopics and Peak accessibility. (**k**) UMAP embedding colored by the transferred cell class from the primary reference. OPC, oligodendrocyte precursor cells; VLMC, vascular and leptomeningeal cells; VSMC, vascular smooth muscle cells. (**l-m**) Heatmap showing consistency between projected labels from the transcriptomic (rows) or chromatin accessibility (columns) primary atlases for the cell class labels (**l**) and region/tissue labels (**m**). (**n**) Heatmap showing average similarity of neuronal subtypes to primary counterparts, based on opened chromatin profiles.

We next analyzed chromatin accessibility dynamics in the multi-omic atlas of posterior brain organoid development. In total, 243,296 genomic regions were detected with significant coverage, and region accessibility profiles show strong heterogeneity across the different time points and cell types (Fig. 2a–d and Extended Data Fig. 4a–c). We performed two parallel analyses to identify gene regulatory regions underlying cell type differentiation. First, we used cisTopic to perform topic analysis on accessibility profiles^29^, identifying 49 clusters of co-accessible regions (regulatory topics) (Fig. 2e). Second, we used a generalized linear model-based differential accessibility test and identified 74,299 differentially accessible regions across cell types. Notably, 21 out of 49 regulatory topics were also significantly differentially accessible (one-sided Fisher’s exact test, Bonferroni-corrected *P* < 0.01) (Fig. 2f–h). Interestingly, we found distinct sets of differentially accessible regions marking different neuron cell types, but did not observe pan-neuronal accessible region clusters, implying that the genome-wide chromatin accessibility dynamics are critical in the specification of distinct neuron cell types. This analysis, coupled with differential expression analysis for the downstream genes, also allows the identification of putative regulatory elements defining the identity of different neuronal cell types (Fig. 2i, Extended Data Fig. 4d–h and Supplementary Tables 4 and 5).

To benchmark how well organoid chromatin accessibility dynamics recapitulate human early brain development, we developed an approach combining topic analysis and transfer learning to project organoid single-cell assay for transposase-accessible chromatin with sequencing (scATAC-seq) data to a single-cell chromatin accessibility atlas of the human developing brain^30^ (Fig. 2j and Extended Data Fig. 4i,j). This allows estimation of which cells of the primary reference are prevalent in organoids, as well as estimation of similarity in chromatin accessibility between organoid and matched reference cells. Organoid cells were matched to primary counterparts, with NPCs, neuroblasts, intermediate progenitor cells and neurons showing high similarities in chromatin accessibility topics (Fig. 2k–m and Extended Data Fig. 4k–n). Transferred cell class and region labels based on transcriptome or chromatin accessibility profiles are largely consistent, indicating that cells in the organoids recapitulate major aspects of transcriptome and chromatin features of primary brain development.

### Inferring and perturbing regulatory programs of human posterior brain organoid development

**Fig. 3:**
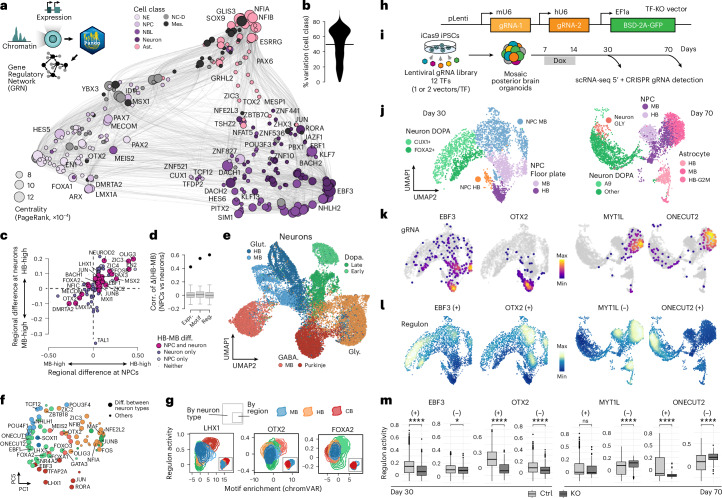
Inferring and perturbing human posterior brain organoid developmental regulomes. **a**, Schematic of GRN inference using the Pando framework (top left); UMAP embedding of the inferred TF network based on co-expression and inferred interaction strength between TFs. Dot color indicates the cell class with the highest expression of the indicated TF, and dot size represents the PageRank centrality of each TF. **b**, Violin plot showing the variation of module activity, explained by cell class. **c**, Comparison of regional differences of the regulon activity for neurons and for NPC. Regulon activities for NPCs and neurons were summed based on regional identity, and the difference between hindbrain (HB) and midbrain (MB) was calculated for each TF. Dot size and color represent differential regulon activity between midbrain and hindbrain: active in both neuron and NPC, in only one or in neither. **d**, Correlation of hindbrain–midbrain differences in TF expression, motif enrichment and regulon activities between NPC and neurons. Boxes show background correlations estimated by permutations of gene or motif labels (*n* = 100). **e**, UMAP embeddings of 24,153 neuronal cells, constructed based on a weighted multimodal neighbors graph, representing a weighted combination of RNA-seq and ATAC-seq modalities. Colors represent neuronal subtypes. **f**, PCA embedding of selected TFs, colored by neuron subtype with the highest expression value of each TF. Dot size indicates whether the regulon is classified as differentially active between neuron subtypes. **g**, Density plot, indicating motif enrichment and regulon activity for selected TFs for each neuron subtype. The small panel in the bottom right represents the same analysis split by the region identity of neurons. **h**, Schematic illustrating a knock-out construct, carrying two gRNAs per gene. **i**, Schematic illustrating the single-cell pooled TF-KO experiment. **j**–**l**, UMAP embeddings of cells with detected gRNA for day 30 (left) and day 70 (right) time points, colored by cell type identity (**j**), detected gRNA density (**k**) and positive (+) or negative (−) regulon activity for each indicated TF (**l**). **m**, Boxplots showing the difference in positive (+) and negative (−) regulon activity between control and KO cells for day 30 organoids (left) and day 70 organoids (right). ****P < 0.0001; ***P < 0.001; **P < 0.01; *P < 0.05; ns, not significant. *n* = 273, 288, 145, 75, 205, 403, 100 and 166, respectively; exact *P* values are 0.046, 1.3 × 10^−15^, 1.8 × 10^−8^, 2 × 10^−^^16^, 6.8 × 10^−^^5^, 0.61, 2.5 × 10^−^^8^ and 4.1 × 10^−^^11^, respectively. Significance was evaluated using a two-sided Wilcoxon rank-sum test, and no adjustment for multiple comparisons was applied.

**Extended Data Fig. 5:**
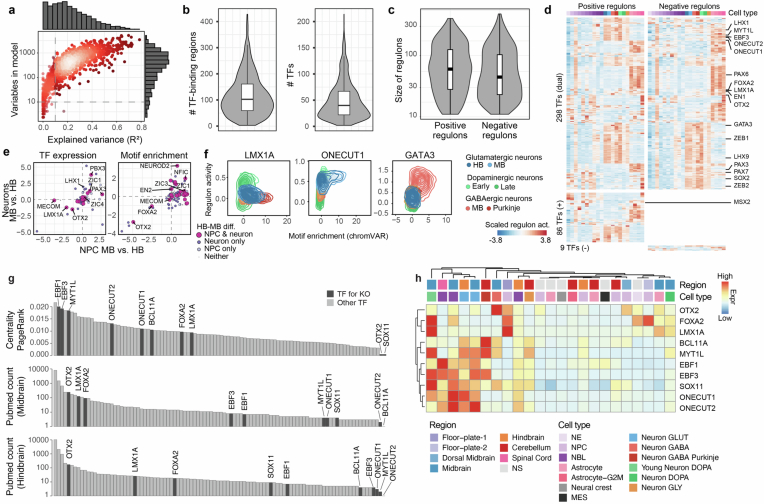
Supplementary analysis of the regulome inference using the PANDO framework. (**a**) Scatter plot and histograms showing explained variance versus number of variables in models used for GRN construction. (**b**) Violin plots showing the distribution of predicted binding regions (left) and TFs (right) per target gene in the GRN (*n=1013*).(**c**) Violin plots showing regulon size (number of genes per TF), split by positive and negative regulons *(n=393 and 308)*. (**d**) Positive and negative regulon activity, averaged by cell type. In total, 298 TFs showed dual activity (acting as repressors for some genes and activators for others), 86 TFs showed only positive activity, and 9 TFs only negative. (**e**) Comparison of regional differences in the TFs expression (left) and motif enrichment in open chromatin (right) for neurons (y-axis) and for NPC (x-axis). Values were summed by regional identity and then the difference between hindbrain and midbrain was calculated for each TF. The size and color of the dots represent the results of the DE analysis of regulon activity between midbrain and hindbrain: if the regulon was in DE for both neuron and NPC, only in one of them, or in neither. MB, midbrain; HB, hindbrain. (**f**) Density plot showing motif enrichment and regulon activity for selected TFs across neuronal subtypes. (**g**) Bar plots showing PageRank regulon centrality (top), the number of papers in which terms TF name and ‘Midbrain’ co-occur (middle) and the number of papers, in which terms TF name and ‘Hindbrain’ co-occur (bottom) for each TF. (**h**) Heatmap showing average expression of TFs, selected for KO experiment, split by cell type. GLUT, glutamatergic; GABA, gabaergic; DOPA, dopaminergic; GLY, glycinergic; NS, non specific.

We used gene regulatory network (GRN) inference to understand how differentiation and specification of different cell types are regulated in human posterior brain organoids^31^. We globally modeled the interaction between TF expression, binding motif accessibility and target gene expression. We identified a GRN involving 393 TFs, accounting for 384 positive (activating) TF regulons and 308 negative (repressive) TF regulons, largely reflecting cell class differences (Fig. 3a,b and Extended Data Fig. 5a–d). Regulons showed significant activity differences between cells with midbrain and hindbrain identities, while NPCs and neurons within a given region showed significantly correlated regulon activities, TF expression and motif enrichment (Fig. 3c,d and Extended Data Fig. 5e). We identified TFs with regulon activities that correlate with neuron cell type identities, including *LHX1* for Purkinje cells, *OTX2* for GABAergic midbrain neurons and *FOXA2* for dopaminergic neurons (Fig. 3e–g and Extended Data Fig. 5f).

**Extended Data Fig. 6:**
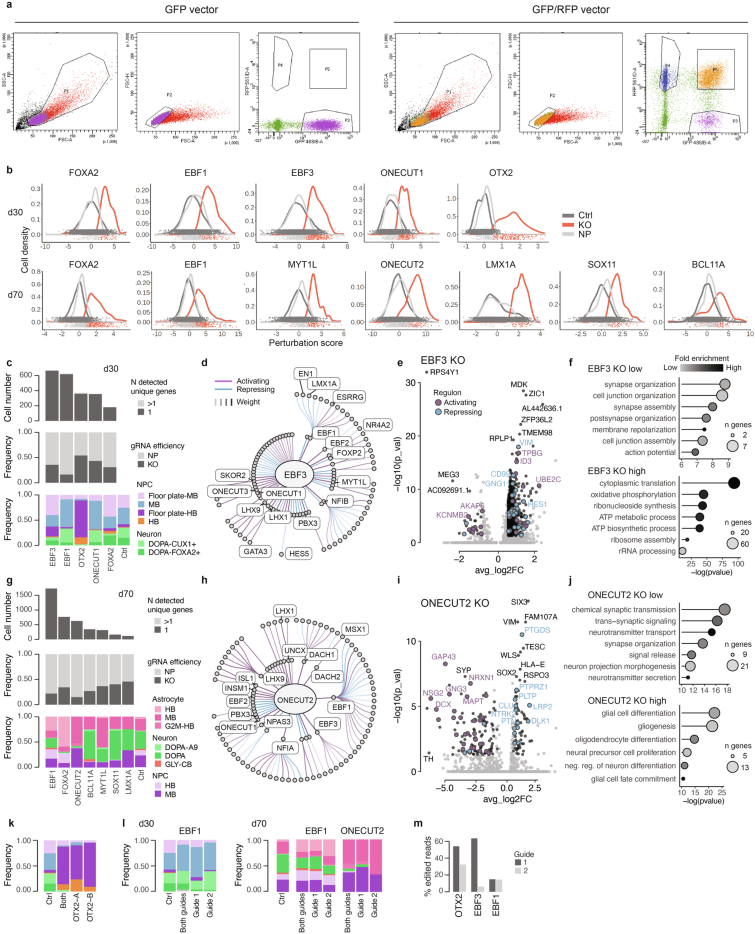
Selection of target genes for single-cell perturbation experiment and supplementary analysis of CRISPR-KO screen data. (**a**) Exemplary fluorescence-activated cell sorting plots of the sorting scheme used to isolate GFP vector positive (left) and GFP/RFP vector positive(right) iPSCs. (**b**) Distribution of perturbations scores for TF-targeting gRNAs, split by guide efficacy classification. Ctrl, non-targeting gRNAs; NP, non perturbed: guide is detected, but not efficient; KO, knock-out. (**c, g**) Bar plots showing, for each target KO gene, the number of cells carrying an efficient guide and the proportion of detected genes per cell (top), the proportion of efficient guides (middle), and the proportions of cell types (bottom) at day 30 (**c**) and day 70 (**g**). MB, midbrain; HB, hindbrain; DOPA, dopaminergic neurons, NP, not perturbed; KO, knock-out. (**d, h**) GRN subgraph for *EBF3* (**d**) and *ONECUT2* (**h**), showing first- and second-order TF targets (nodes). Edges are colored based on TF regulatory interaction: purple is activating, blue - repressing. (**e, i**). Volcano plots showing DE between *EBF3*-KO and control cells for day 30 time point (**e**) and between *ONECUT2*-KO and control cells for day 70 time point. Significance was assessed using a two-sided Wilcoxon rank-sum test, and p values were adjusted for multiple comparisons using Bonferroni correction. (**i**). DEGs are colored in black. DEGs belonging to positive and negative regulons are further labeled in purple and blue, respectively. (**f, j**) GO analysis or DE gene sets identified in e and i. Rows represent GO terms, x-axis shows p-values and dot size is proportional to gene set size. Significance for each GO term was assessed using one-sided Fisher’s exact test for over-representation. p values were adjusted for multiple comparisons using the Benjamini–Hochberg false discovery rate method. (**k**) Bar plots showing cell type proportions for control cells and cells carrying guides from individual lentiviral vectors (OTX2-A and OTX2-B) and both vectors for *OTX2*-KO, day 30 organoids. (**l**) Bar plots showing cell type proportions for control cells and cells with either individual or combined guides from *EBF1*-KO at day 30 (left) and from *EBF1*-KO and *ONECUT2*-KO at day 70 (right). (**m**) Bar plots showing the percentage of edited reads for *OTX2*-KO, *EBF3*-KO and *EBF1*-KO for each guide.

We used a pooled, multiplexed CRISPR–Cas9 gene knock-out (KO) experiment with single-cell transcriptomic readout to perturb and validate selected TF regulomes (Fig. 3h). We designed guide RNAs (gRNAs) and generated a pooled lentiviral library targeting 12 TFs with specific midbrain or hindbrain expression or high regulatory centrality (Extended Data Fig. 5g,h and Supplementary Table 6). Those iPS cells carrying a doxycycline-inducible *Cas9* cassette in the AAVS1 safe-harbor locus were transduced, mosaic organoids were generated and perturbations were induced at the neuroepithelium stage from day 7 to day 14 (Fig. 3i). Mosaic organoids were analyzed using scRNA-seq and gRNA amplicon sequencing at day 30 and day 70, recovering 31,857 cells, among which a gRNA was detected in 8,711 cells (Fig. 3j and Extended Data Fig. 6a–j).

We observed differential cell type abundance associated with specific perturbations in day 30 organoids. For example, perturbation of *OTX2*, a well-known midbrain fate regulator^6^, resulted in a complete shift in cell type composition towards hindbrain regions, with the result being consistent across two different lentiviral constructs (Fig. 3k and Extended Data Fig. 6c,k–m). *OTX2* regulon activity was highest in midbrain cells (Fig. 3l), with the regulon suppression activity in KO cells (Fig. 3m). As another example, *EBF3* perturbation resulted in an enrichment of NPCs, consistent with regulon activity in neurons (Fig. 3k–m). *EBF3* is inferred to regulate the expression of numerous neural differentiation genes, including *MYT1L, ONECUT1* and *ONECUT2* (Extended Data Fig. 6d,e and Supplementary Table 7). Differential gene expression analysis between *EBF3*-KO and control cells revealed that downregulated genes were enriched in synapse organization, assembly and membrane repolarization functions (Extended Data Fig. 6f). This finding aligns with the shift in cell type composition observations and suggests that *EBF3* has an important role in the transition from NPC to neuron by activating key neural differentiation genes, possibly explaining developmental disruption resulting from EBF3 mutation^32^.

At day 70 of organoid development, we observed the increased prevalence of *MYT1L*-KO cells in astrocytes, which is in concordance with the well-known role of this gene in neurogenesis, suppressing the differentiation of non-neuronal fates (Fig. 3j and Extended Data Fig. 6g). Furthermore, we observed increased activity of *MYT1L* negative regulons in the KO cells (Fig. 3l,m), which aligns with *MYT1L* acting primarily as a repressor^33^. *ONECUT2* is another gene in which perturbation disrupts NPC and neuron abundance (Fig. 3l,m), with perturbed cells depleted in neurons and enriched in astrocytes. Genes upregulated in *ONECUT2*-KO cells are related to gliogenesis, whereas downregulated genes are related to synapse transmission, neurotransmitter transport and secretion (Extended Data Fig. 6h–j and Supplementary Table 7), which is consistent with the role of *ONECUT2* as a neural fate inductor^34^. Together, these data provide a multiplexed single-cell perturbation experiment in organoids to examine genetic regulation of posterior cell fate specification and reveal underlying mechanistic pathways.

### Combinatorial morphogen screen introduces a spectrum of organoid models of the posterior human brain

**Fig. 4:**
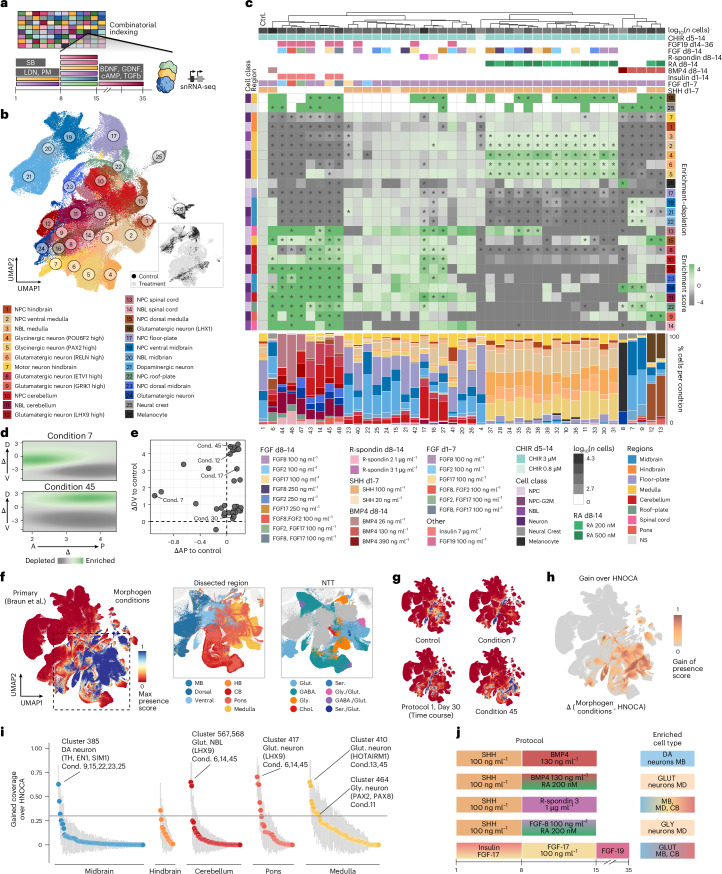
A combinatorial morphogen screen expands posterior organoid protocols. **a**, Experimental scheme and timeline of the combinatorial morphogen patterning screen with single-cell transcriptomics readout. A total of 96 individual organoids, each exposed to one of 43 morphogen combinations, were individually analyzed with snRNA-seq using split-pool combinatorial barcoding (Parse Biosciences). **b**, UMAP embedding of 177,718 cells in the dataset, colored by cluster identity (left) and mode of treatment (bottom right). **c**, Overview of conditions and results of combinatorial morphogen screen. Heatmap showing enrichment–depletion scores (log_2_ of cell type proportions relative to control), with *P < 0.05 (false discovery rate-adjusted using the Benjamini–Hochberg procedure). Each column represents a distinct morphogen combination, with color-coded annotation shown at the top of the column. Each row corresponds to a cluster with color-coded annotation: left side, cell class and region; right side, cell types, corresponding to those shown in the UMAP embeddings in **b**. Stacked barplots (bottom) show the relative composition of organoids per condition; condition names are labeled at the bottom. Morphogen conditions and cell types were clustered hierarchically based on enrichment–depletion values. The control condition (label 1) was not included in the clustering and was added later for visualization. NS, non-specific; Ctrl., control. Detailed information about statistical tests is described in the Methods (‘Determination of composition changes in morphogen perturbation screen’). **d**, Visualization of difference in spatial mapping of selected morphogen treatment conditions compared with control using the dorso-ventral (vertical) and anterior–posterior (horizontal) axis of developing human brain radial glia^28^. D, dorsal; V, ventral; A, anterior; P, posterior. **e**, Scatterplot showing the aggregated location of each morphogen screen condition along the DV and AP axis relative to control. Cond., condition. **f**, UMAP of the primary reference, colored by the max presence scores across all screen conditions. The area indicated with a black outline is further colored by dissected regions (center) and neural transmitter transporter (right). CB, cerebellum; NTT, neural transmitter transporter; Glut., glutamatergic; GABA., gabaergic; GLY., glycinergic; Chol., cholinergic; Ser., serotonergic. **g**, UMAP of primary reference, colored by max presence scores across the indicated screen conditions and protocol 1 day 30 time point from the posterior brain organoid atlas. **h**, UMAP of the primary reference, colored by gained coverage of posterior brain organoid morphogen screen over the human neural organoid cell atlas (HNOCA)^22^, with negative values trimmed to zero. **i**, Gain of cell cluster coverage of the screen conditions over HNOCA, corresponding to the average max presence scores per primary reference cluster, with negative values trimmed to zero. The threshold is set to 0.3 and represented by a gray horizontal line. Circles show mean cluster gain and are colored by the most abundant dissected region of the cluster; gray vertical lines indicate the standard deviation of gain coverage. Cell cluster sizes correspond to those described in the original publication^28^. **j**, Schematic showing the protocols, tested in the morphogen screen, allowing for enrichment of the representative cell types.

Posterior cell type diversity is underrepresented in current brain organoid protocols^22^. We devised a screen to systematically evaluate whether morphogen combinations can induce a new spectrum of posterior neuron subtypes (Fig. 4a). We based the screen on protocol 1 including neural induction using dual SMAD inhibition during the first 7 days of organoid culture, and varied exposure to morphogen combinations in different time windows with a major focus on the second week of organoid development during initial neuronal differentiation (Fig. 4a). We chose known modulators of AP patterning (the WNT signaling agonist CHIR; RA; FGF8, FGF2 and FGF17; R-spondin 2 and 3) as well as dorso-ventral (DV) patterning (BMP4; insulin; SHH; SHH pathway agonist purmorphamine) and designed 48 distinct morphogen modulator conditions across a range of concentrations and combinations (Supplementary Table 8).

**Extended Data Fig. 7:**
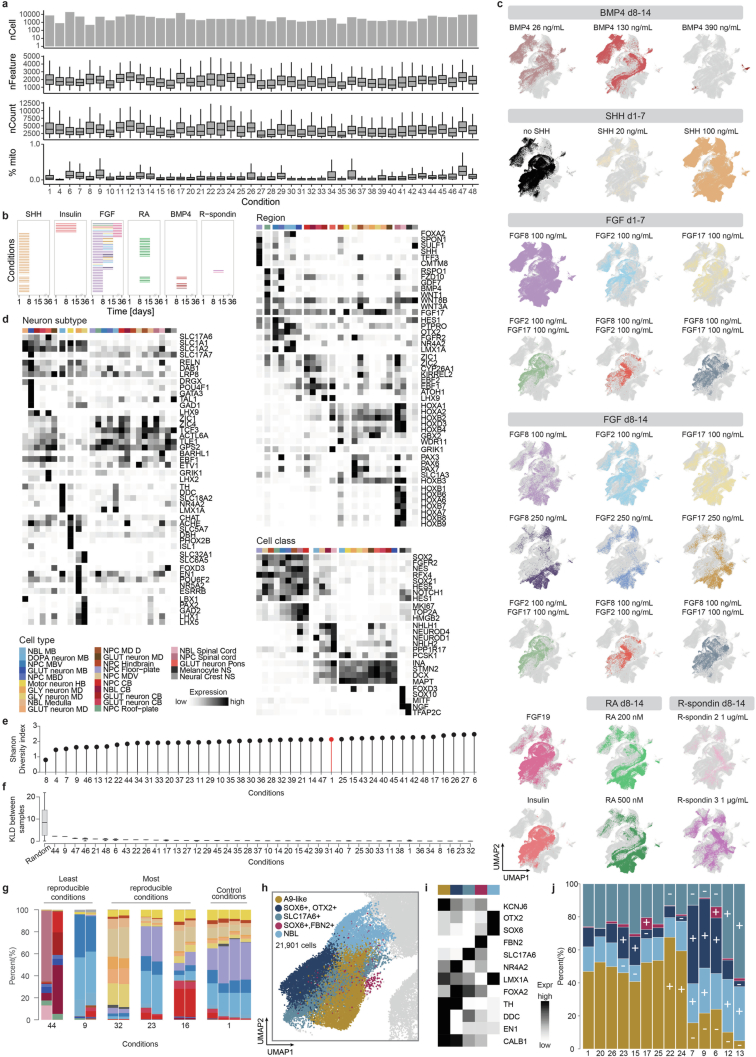
Supplementary analysis of morphogen screening experiment. (**a**) Quality control metrics for the morphogen screen snRNA-seq. Each column represents a treatment condition. nCell, number of cells; nCount_RNA, total number of RNA molecules detected within a cell; nFeature_RNA, total number of genes detected within a cell; %mito, percentage of mitochondrial genes; nCount, number of detected reads. Exact sample sizes could be found in Supplementary Table 1. (**b**) Representations of morphogen timings in all conditions. (**c**) UMAP embeddings coloured by morphogen treatment. For each panel, treated cells are shown in color and all other cells in gray. (**d**) Heatmaps showing min.-max. scaled mean expression of marker genes across cell types. Genes are grouped by cell class markers (top left), region markers (right), neuronal subtype markers (bottom left). (**e**) Lollipop plot showing the Shannon diversity index for each condition, calculated from cell type proportions. Control condition is highlighted in red. (**f**) Box plots showing pairwise Kullback–Leibler divergence between organoid replicates and matched morphogen conditions. The first barplot ‘Random’ shows a control KLD distribution between 50 randomly paired organoid samples. Lower values indicate higher reproducibility. KLD, Kullback–Leibler divergence. Exact sample size for each condition could be found in Supplementary Table 1. (**g**) Stacked bar plots showing cell type distributions across replicates for the most and least reproducible conditions, and control organoids. Legend is the same as in panel (**d**). (**h**) UMAP embedding showing cells selected for downstream dopaminergic neuron analysis colored by dopaminergic neuron subtypes. (**i**) Heatmaps showing min-max scaled mean expression of marker genes for each cell type. (**j**) Stacked barplot showing the distribution of dopaminergic neuron subtypes in conditions with >300 detected cells. Subtypes significantly enriched or depleted in a given condition are indicated with ‘+’ or ‘–’, respectively (FDR-adjusted p < 0.05, Benjamini–Hochberg correction). Detailed information about statistical test is described in Methods, Determination of composition changes in morphogen perturbation screen.

Organoids were grown under 48 different morphogen conditions, differentiated until week 5 and individually dissociated for single-nucleus RNA-seq (snRNA-seq) profiling using combinatorial barcoding. Notably, five out of the 48 conditions resulted in organoids with reduced size and were excluded from snRNA-seq profiling. In total, 177,718 high-quality cells were profiled across 43 conditions, with a median of 2,798 cells and two individual organoid replicates per condition (Extended Data Fig. 7a–g). Clustering of the single-cell transcriptomic data revealed 25 molecularly distinct cell types, with strong cell type abundance changes dependent on morphogen treatments, particularly for RA and BMP4 (Fig. 4b and Extended Data Fig. 7d). Cell type composition across individual organoids for a given condition was highly reproducible (Extended Data Fig. 7f,g).

Based on organoid cell type composition, we classified morphogen effects into at least five groups (Fig. 4c and Supplementary Table 9): lack of SHH or insulin generated glutamatergic neurons from midbrain, cerebellum and pons, which is expected in the absence of ventralizing signals; BMP4 inclusion at an intermediate dose resulted in an increased proportion of ventral midbrain progenitors and dopaminergic neurons (clusters 20, 21), which was previously described in 2D cultures context^35^; RA conditions resulted in a variety of ventral medulla cell types (clusters 1–7), including two subtypes of glycinergic neurons; the combination of BMP4 and RA led to the emergence of neuronal cells of the dorsal medulla (clusters 15, 16), revealing additive effects combining posterior and dorsal stimuli; and both R-spondins, as well as BMP4 at a low dose, gave rise to a diverse range of midbrain, cerebellum and medulla cell types. A Shannon diversity index calculation (Extended Data Fig. 7e) was used to represent the conditions with the highest cell type diversity.

Significant shifts in the composition of dopaminergic neuron subtypes were also observed in treatment conditions in comparison to the control (Extended Data Fig. 7h–j). The administration of R-spondin 3 and a low dose of BMP4 resulted in an increased proportion of *FBN2*^+^ dopaminergic subtypes, whereas FGF2 in low dose (100 ng ml^−1^), provided early and late, resulted in the increased proportion of A9-like dopaminergic neurons. The administration of BMP4 at an intermediate dose alone increased the proportion of *SOX6*^+^*OTX2*^+^ dopaminergic neurons, while its combination with RA led to the emergence of a greater proportion of *SLC17A6*^+^(*Vglut2*^+^) dopaminergic neurons in a RA dose-dependent manner.

**Extended Data Fig. 8:**
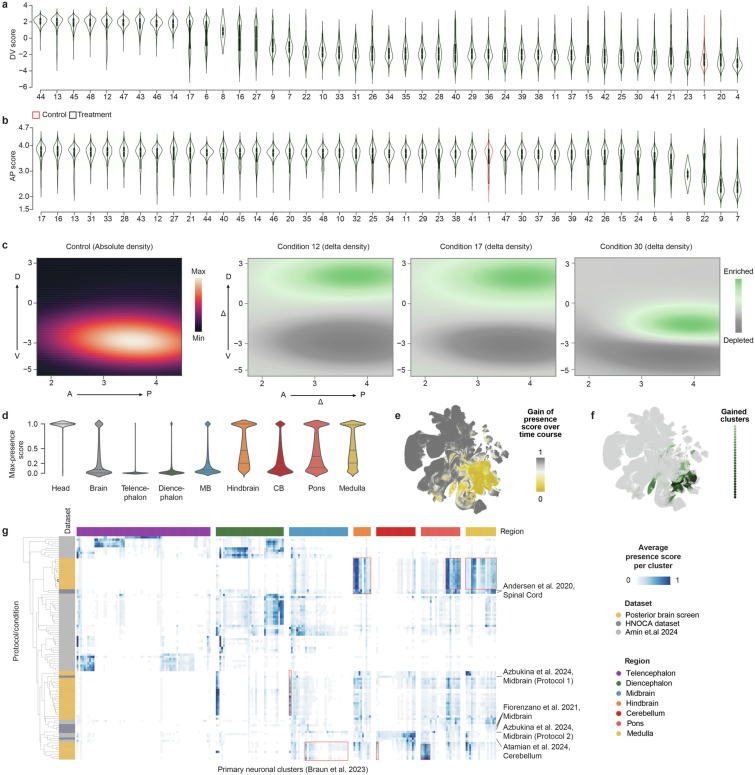
Supplementary analysis of mapping morphogen screen experiment to primary human brain atlas. (**a, b**) Violin plots showing distribution of dorsal-ventral (**a**) and anterior-posterior (**b**) scores for each condition, ordered by descending median. The control condition is highlighted in red. DV, dorsal-ventral; AP, anterior-posterior. Exact sample sizes could be found in Supplementary Table 1. (**c**) Density plot showing the distribution of AP and DV scores for each cell in the control condition (left). Visualization of spatial mapping differences for selected morphogen treatment conditions compared with control, along the dorsal–ventral (vertical) and anterior–posterior (horizontal) axes of developing human radial glia^28^. D, dorsal; V, ventral; A, anterior; P, posterior. (**d**) Violin plots showing the distribution of max presence scores of morphogen screen data in primary data, stratified by dissected regions of the primary brain atlas. (**e**) UMAP of the primary reference, colored by gained coverage of the posterior brain organoid morphogen screen over posterior brain organoid time course atlas with negative values trimmed to zero. (**f**) UMAP of the primary reference, colored by gained cell types clusters in the morphogen screen data. (**g**) Heatmap showing posterior morphogen screen data with selected HNOCA datasets, generating posterior brain cell types and conditions from the published screen data (Amin et al. ^24^) on the basis of average presence scores of neuronal clusters in the primary reference. The heatmap shows average presence scores per cluster in the primary reference (columns).

To quantify the influence of morphogen conditions on DV and AP fate identity, we calculated DV and AP scores for each sequenced organoid progenitor cell using a regularized linear model, trained on the radial glia of the human developing brain cell atlas^28^. Subsequently, for all progenitors in a given treatment condition, differential AP and DV scores relative to the control were calculated (Fig. 4d,e and Extended Data Fig. 8a–c). Condition 7, for instance, exhibits a slight dorsal and anterior shift relative to the control, resulting in a high proportion of midbrain dopaminergic neurons. By contrast, condition 45 displays a dorsal and posterior shift, predominantly comprising cell types with cerebellar identity.

We mapped the morphogen screening data to a primary human developing brain cell atlas^28^ using transfer learning to assess the correspondence to primary counterparts. We estimated presence scores for every primary cell type in each morphogen screen condition^22^ and found that screen conditions were enriched for midbrain and hindbrain region identities, while telencephalic or diencephalic cell types were absent (Fig. 4f and Extended Data Fig. 8d–g). Notably, the presence scores of control organoids from the screen are consistent with those of organoids from our previous time-course dataset (Fig. 4g), indicating robustness of the organoid protocol.

**Extended Data Fig. 9:**
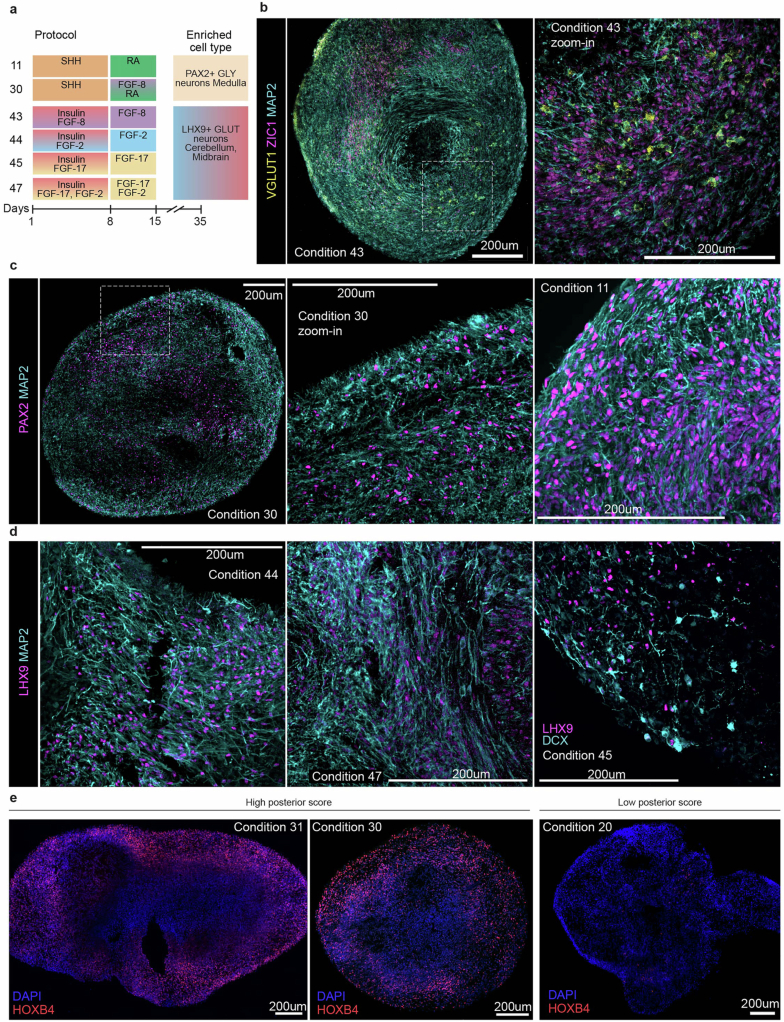
Validation of neuronal subtypes identified in the morphogen screen. (**a**) Schematic showing conditions tested in the morphogen screen, allowing for enrichment of the representative cell types. (**b**) Immunofluorescence staining of VGLUT1, MAP2 and ZIC1 marking cerebellar glutamatergic neurons. (**c**) Immunofluorescence staining of PAX2 and MAP2 marking medullary PAX2+ neurons. (**d**) Immunofluorescence staining of LHX9 and MAP2/DCX, marking LHX9+ neurons. (**e**) Immunofluorescence staining of HOXB4 showing increased expression in organoids with higher posterior score.

We next evaluated whether the morphogen screen conditions generated cell states previously absent or underrepresented in in vitro organoid protocols. We therefore compared the max presence scores of the morphogen screen data to those estimated for the integrated human neural cell organoid atlas (HNOCA)^22^. This analysis revealed that cell types from midbrain and hindbrain regions were enriched in the screen (Fig. 4h), with a total of 28 reference cell clusters showing a significant abundance increase in certain treatment conditions (Fig. 4i). The most enriched clusters included dopaminergic neurons from the ventral midbrain, *LHX9*^+^ glutamatergic neurons in the cerebellum and pons, and *PAX2*^+^/*PAX8*^+^ glycinergic neurons in the medulla. These newly detected cell types were validated using immunofluorescence microscopy (Extended Data Fig. 9a–e). In addition to identifying specific enriched clusters, we compared cell type presence scores from the morphogen screen to those from existing posterior organoid protocols, including HNOCA and a previously published morphogen screen^24^ (Extended Data Fig. 8g).

In summary, the morphogen screen expanded posterior brain cell type diversity generated with in vitro organoid cultures and serves as a resource to fine-tune organoid protocols (Fig. 4j).

### Differential abundance-informed trajectory inference provides inroads to understanding human brain development

**Fig. 5:**
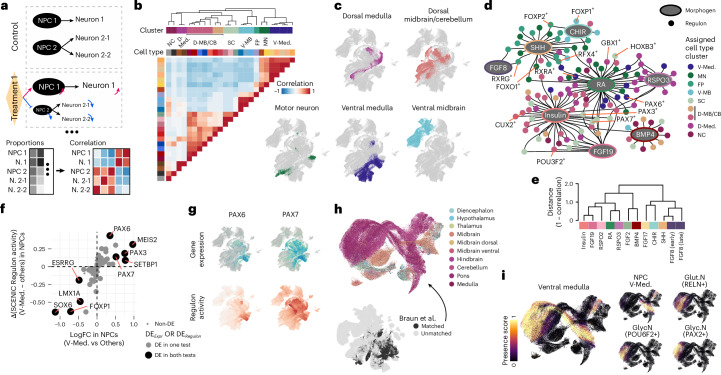
Perturbation-informed trajectory inference reveals regulatory mechanisms underlying neural differentiation trajectories. **a**, Schematic of differentiation trajectory inference based on cell type compositions across multi-treatment experiments. **b**, Hierarchical clustering of cell types from morphogen screening based on pairwise correlation of cell type proportions across morphogen conditions (heatmap). NC, neural crest; D-Med., dorsal medulla; D-MB, dorsal midbrain; SC, spinal cord; V-MB, ventral midbrain; FP, floor plate; MN, motor neuron; V-Med., ventral medulla. **c**, UMAPs of five differentiation trajectories for distinct neuronal cell types identified through composition-based clustering. **d**, Morphogen–regulon regulatory networks identified by GRNBoost2. **e**, Hierarchical clustering of morphogen treatments based on their importance in activating or inhibiting regulon activities. **f**, Differential expression of TFs and differential activity of their SCENIC-reconstructed regulons between ventral medulla NPCs and other NPCs. **g**, Expression and regulon activity UMAPs; for example, TFs PAX6 and PAX7. **h**, UMAP of the human developing brain cell atlas. Bottom, cells matched to morphogen screening cell types (matched primary sub-atlas); top, matched primary sub-atlas colored by dissected brain regions. **i**, Matched primary sub-atlas UMAP showing presence within ventral medulla cell clusters from morphogen screening.

**Extended Data Fig. 10:**
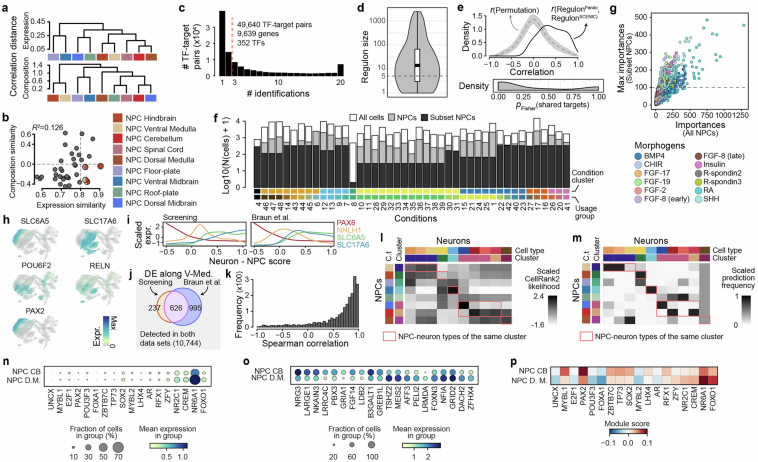
Supplementary analysis of trajectory analysis and inference of regulatory mechanism by morphogens. (**a**) Dendrograms showing similarities between NPC cell types based on gene expression distances (top) or co-occurrence distances (bottom). (**b**) Correlations between expression and co-occurrence similarities. Each dot represents one pair of NPC cell types. Dashed lines indicate thresholds to define high and low similarities. Pairs with high expression similarities but low co-occurrence similarities are shown as two semicircles, each of which is colored by a NPC cell type of the pair. (**c**) Frequency of TF–target pairs identified across 20 independent SCENIC runs using random subsets of NPCs. (**d**) Distribution of regulon sizes, that is numbers of predicted targets per TF (n=352). The dashed line marks the size threshold for the final regulon list. (**e**) Consistency of regulon inference between the SCENIC results and the previous Pando results. The upper panel shows distribution of correlation between regulon activities estimated based on the SCENIC prediction and the previous Pando-based inference. The area in shade shows the distribution of 100 permutations (±1SD). The lower panel shows the one-sided Fisher’s exact test p-value distribution for overlap of predicted targets per TF with the two approaches. P-value smaller than 0.5 indicates more overlap than expected. (**f**) Numbers of all cells, all NPCs, and targeted NPCs in each condition used by GRNBoost2 to infer morphogen–regulon relationships. (**g**) Correlation between estimated importances for morphogen–regulon pairs inferred by GRNBoost2 using either all NPCs (x-axis) or maximum values across 50 random subset runs (y-axis). Dots are colored by morphogen identity. (**h**) UMAP of the matched subset of primary atlas to the screening data, colored by selected markers of the ventral medulla cell types. (**i**) Expression profiles of selected marker genes across ventral medulla NPC-neuron differentiation scores in the screening data (left) and the primary data (right). (**j**) Numbers of differentially expressed genes (DEGs) along the ventral medulla differentiation in the screening data and the primary atlas, and their overlap. (**k**) Distribution of Spearman correlations between differentiation-dependent expression profiles of the identified DEGs in the two systems. (**l-m**) Scaled differentiation likelihood of NPC populations towards different neuron cell types in the primary atlas, inferred using CellRank2 (**l**) or stepwise elastic net prediction along the differentiation trajectories (**m**). (**n**) Dotplot showing expression of regulon-associated transcription factors in NPC Cerebellum (CB) and Dorsal Medulla (D.M.) clusters. (**o**) Dotplot of the top 20 differentially expressed markers between NPC CB and NPC D.M. populations, illustrating poor separation of these populations at the transcriptomic level. (**p**) Heatmap showing average regulon activity in NPC Cerebellum (CB) and Dorsal Medulla (D.M.) clusters for selected differentially active regulons.

The shared differential abundance of progenitor and neuronal populations across morphogen patterning conditions can inform their relation within a differentiation trajectory (Fig. 5a). We calculated pairwise Pearson correlation (*r*) between proportions of cell types across conditions, and hierarchically clustered cell types using the correlation distance (defined as 1 − *r*) matrix (Fig. 5b). This allowed the identification of nine cell type clusters, which could be summarized into five distinct NPC-neuron trajectories (Fig. 5c). Importantly, the annotated regional identity of NPC and neuron populations assigned to the same trajectory matched, such as ventral midbrain NPCs, neuroblasts and dopaminergic neurons assigned to the ventral midbrain trajectory, or ventral medulla NPCs, medulla neuroblasts, glycinergic neurons and *RELN*-high glutamatergic neurons assigned to the medulla trajectory. Notably, although the shared differential abundance of NPC populations can be positively correlated with their transcriptomic similarities (Extended Data Fig. 10a,b), there are cases in which NPC populations with high transcriptomic similarity are exclusively found across conditions, indicating that transcriptomic similarity can mislead trajectory inference.

To explore the gene regulatory logic underlying the diversification of NPC pools in the presence of morphogen cues, we performed a hierarchical regulatory network reconstruction analysis as previously described^36^. In brief, we identified TF regulons in the screening data using SCENIC (Extended Data Fig. 10c–g) and inferred their regulatory relationship with morphogens using GRNBoost2. This analysis linked morphogens to different sets of regulons that showed distinct activity patterns along the different neuronal differentiation trajectories (Fig. 5d). For instance, RA was linked to many regulons with enriched activities in the ventral medulla (for example, *PAX6*^+^, *RXRA*^+^) and dorsal medulla (for example, *GBX1*^+^) trajectories, indicating the essential role of RA in activating medulla-specific genetic programs. Some regulons showed linkage to multiple morphogens, such as *PAX7*^+^, which was activated by both RA and insulin. Notably, the relationship between RA together with PAX6 and PAX7 has been previously described only in the context of spinal cord^37^. Based on the inferred regulatory weights and directions for morphogens on different regulons, we were also able to evaluate the similarities in the influence of of different morphogens on neuron cell fate determinations (Fig. 5e). Focusing on the ventral medulla trajectory, we identified *PAX6* and *PAX7* as critical fate regulators linked to RA and with enriched expression and regulon activities in ventral medulla NPCs compared to other NPC populations (Fig. 5f,g). In addition, TFs such as *SOX6*, *FOXP1* and *LMX1A* showed negative correlation with ventral medulla identity, suggesting that these TFs and their regulons need to be turned off in order to adopt ventral medulla fate.

To assess the in vivo relevance of the identified differentiation trajectories, we subsetted the human developing brain cell atlas^28^ to cells confidently mapping to the organoid morphogen screening data (Fig. 5h). Focusing on primary NPCs and neurons matching the organoid ventral medulla cells (Extended Data Fig. 10h), we could reconstruct a trajectory from NPCs towards three different neuronal populations: *RELN*-high glutamatergic neurons, *POU6F2*-high glycinergic neurons and *PAX2*-high glycinergic neurons (Fig. 5i and Extended Data Fig. 10i), consistent with the organoid ventral medulla trajectory. Pseudotemporal gene expression dynamics in the organoid data showed high similarity to the primary ventral medulla trajectory (Extended Data Fig. 10j,k). Finally, we compared the performance of differential abundance-informed trajectory inference to that of conventional transcriptome-similarity-based trajectory inference methods^38^. Although certain regional differentiation trajectories could be readily inferred from the primary brain atlas data using transcriptome-similarity alone (for example, dorsal midbrain), others failed, probably owing to high transcriptomic similarity to progenitor populations from other brain regions (for example, dorsal medulla) (Extended Data Fig. 10l,m). In those cases, information about differential abundance in the organoid screening data provided important additional information to resolve trajectories. For example, dorsal medulla and cerebellum NPCs are transcriptomically similar, lacking clear markers that distinguish these two populations (Extended Data Fig. 10n,o). However, these populations clearly separate using differential abundance across morphogen conditions and show distinct regulon activity signatures (Extended Data Fig. 10p). Altogether, these data highlight that systematic in vitro organoid morphogen perturbations enable trajectory inference across brain regions.

## Discussion

We present a thorough, scMultiome atlas of human posterior brain organoid development. We used two existing protocols originally developed to generate midbrain cell types and found that both protocols generate significant portions of hindbrain cells in addition to midbrain identities. Similar observations were made for other midbrain protocols^39^, indicating that further efforts are required to achieve higher regional specificity in in vitro cultures and highlighting the importance of organoid protocol profiling at the single-cell level for comprehensive benchmarking and validation of cell type diversity.

To provide insights into the mechanisms underlying human brain regionalization, we complemented the scMultiome atlas with genetic perturbation datasets. We have identified brain region-specific programs, many of which include well-known master regulators, such as *OTX2, LMX1A, LHX1, EN2* and *ZIC3*. Interestingly, the majority of programs are shared between NPCs and neurons on the level of regulon activity, TF expression and motif enrichment, implying that similar genetic programs are responsible for regional identity establishment and maintenance and providing evidence for a deterministic specification of neural diversity^40^.

We use systematic modulation of morphogen signaling pathways to expand the diversity of in vitro-generated cell types of the posterior human brain. The approach generated underrepresented brain cell types and identified protocols that yield cerebellum-specific and pons-specific *LHX9*^+^ glutamatergic neurons as well as glutamatergic and glycinergic neuron types from the dorsal and ventral medulla, respectively. The newly derived cell types could serve as valuable models for hyperekplexia-like disorders^41,42^ (glycinergic neurons, expressing *GLRA1*, *GLRB* or *SLC6A5* genes^43^) as well as for studying the underlying mechanisms of autism spectrum disorders (glycinergic neurons, expressing *PAX2* (ref. ^44^) or *GLRA2* (ref. ^45^) and cerebellar glutamatergic neurons, expressing *GRM5*, *UBE3A*, *GRIA1* and *GRIA2* (refs. ^46,47^)). An additional key application of these posterior brain models is the study of pediatric brain tumors, enabling the evaluation of targeted therapeutics for known driver genes such as *ACVR1* (ref. ^48^), *BRAF*, *NF1* (ref. ^49^) and *PPM1D*^50^, as well as the discovery of unknown drivers contributing to tumor initiation and progression. The screen shows how combinations of morphogen modulators influence the regional identity along the DV and AP axes, as illustrated by BMP4 and RA. BMP4 alone has a dorsalizing effect, resulting in the enrichment of *Vglut2*^+^ dopaminergic neurons, whereas RA alone stimulates the production of posterior ventral cell types. The combination of RA and BMP4 results in the generation of dorsal posterior cells. Notably, RA effects were generally dominant, overriding the effects of various FGFs, suggesting that milder caudalization agents, such as CHIR or lower concentrations of RA, could be used to achieve a higher degree of granularity in brain stem patterning.

We incorporated differential cell type abundance into trajectory inference and reconstructed differentiation events within the morphogen screen data. This approach was possible owing to the unique combination of both progenitor and neuron diversity across morphogen screen conditions and the time point selected for sequencing, as compared to analyzing organoids at earlier time points^23–25^. As an example, dorsal and ventral medulla trajectories were exclusive in different conditions in the morphogen screen data; however, dorsal and ventral medulla NPCs showed high transcriptomic similarity in both the screen data and the primary atlas, making it difficult to resolve differentiation trajectories purely using transcriptomic information. This highlights how organoid morphogen screening data can inform the reconstruction of differentiation trajectories in primary atlases in cases where transcriptome-based methods fail.

Several important limitations should be considered when interpreting these findings. Transcriptomic readouts of genetic and signaling screens do not explicitly indicate how the functions of the neurons are affected. In future studies, the implementation of multiplexed protein staining or functional assays, such as calcium imaging readouts, is expected to provide further biological insights and complement the findings. Altogether, our data demonstrate the potential of organoids to recapitulate primary human posterior brain development at both transcriptomic and chromatin levels. Furthermore, they reveal extraordinary cellular diversity in the posterior brain and provide an integrated overview of morphogen-induced regionalization in posterior brain organoids. These morphogen screens, coupled with single-cell genomic readouts, present opportunities to optimize region-specific protocols and explore neuronal differentiation trajectories with high resolution.

## Methods

### Experimental methods

#### Stem cell and organoid culture

We used three human iPS cell lines (HOIK1, WIBJ2 from the HipSci resource^43^ and WTC from the Coriell Institute). Stem cell lines were cultured in mTeSR Plus with mTeSR Plus supplement (STEMCELL Technologies, 100-0276) and supplemented with penicillin–streptomycin (1:200; Gibco, 15140122) on Matrigel-coated plates (Corning, 354277). After splitting with TrypLE (Gibco, 12605010) or EDTA in DPBS (final concentration, 0.5 mM) (Gibco, 12605010), cells were passaged once or twice per week. Rho-associated protein kinase (ROCK) inhibitor Y-27632 (final concentration, 5 μM; STEMCELL Technologies, 72302) was provided on the first day after passage. Cells were tested for mycoplasma infection regularly using PCR validation (Venor GeM Classic, Minerva Biolabs) and found to be negative. A total of 10,000 cells were plated in ultra-low-attachment plates (Corning, CLS7007) to generate brain organoids using midbrain organoid differentiation protocols^14,15^.

#### Single-cell multiome experiments for the developmental time course

Brain organoids were generated from three different stem cell lines (WTC, WIBJ2 and HOIK1) simultaneously. Brain organoids of the same batch were dissociated at multiple time points of the course of brain organoid development: neural induction (day 7) and neural differentiation and maturation (days 15, 30, 60, 90 and 120). Organoids of the three different cell lines were pooled on the basis of size and dissociated together, and the cell lines were later demultiplexed on the basis of the single-nucleotide polymorphism information. Multiple organoids of each line were pooled together to obtain a sufficient number of cells. If needed, at the later time points, organoids were cut in half and washed three times with HBSS without Ca^2+^ and Mg^2+^ (STEMCELL Technologies, 37250). Tissue dissociation to a single-cell suspension was done with a papain-based dissociation kit (Miltenyi Biotec, 130-092-628). Pre-warmed papain solution (2 ml) was added to the organoids and incubated for 15 min at 37 °C. This step was followed by the addition of enzyme mix A, and then the tissue pieces were triturated five to ten times with 1,000 μl wide-bore and P1000 pipette tips. After that, the tissue pieces were incubated twice for 10 min at 37 °C with trituration steps with P1000 and P200 pipette tips. After dissociation, cells were filtered with 30 μm filters and centrifuged. Before nuclei isolation, 100,000 cells were washed twice with 50 μl PBS containing 0.04% BSA. To isolate nuclei from the ready single-cell suspension, cells were resuspended in 50 μl lysis buffer (10 mM Tris-Cl pH 7.4, 10 mM NaCl, 3 mM MgCl_2_, 1% BSA, 0.1% Tween-20, 1 mM dithiothreitol, 1 U μl^−1^ RNase inhibitor (Roche Protector RNase-Inhibitor), 0.1% NP-40, 0.01% digitonin (Invitrogen, BN2006)) and incubated for 3 min on ice, neutralized by adding 50 μl wash buffer (10 mM Tris-Cl pH 7.4, 3 mM NaCl, 10 mM MgCl_2_, 1% BSA, 0.1% Tween-20, 1 mM dithiothreitol, 1 U μl^−1^ RNase inhibitor). Single-nuclei suspension generation and library preparation were performed according to the 10x Chromium Single Cell Multiome ATAC + Gene Expression kit protocol.

#### Immunofluorescent imaging

Organoids were fixed in 4% PFA at 4 °C overnight, followed by incubation in 30% sucrose solution for 24–48 h. Afterwards, organoids were transferred to plastic cryomolds (Sakura) and embedded in Finetek Tissue-Tek OCT Compound (Sakura) for snap-freezing on dry ice. For immunohistochemical stainings, organoids were sectioned into 20 μm-thick slices using a cryostat (Thermo Fisher Scientific, Cryostar NX50). Organoid sections were quickly washed in PBS to remove any residual OCT. The sections were then incubated in antigen-retrieval solution (HistoVT One, Nacalai Tesque) at 70 °C for 20 min. Excess solution was washed away with PBS, and the tissue was incubated in blocking–permeabilizing solution (0.1% Triton X-100, 0.2% Tween-20 and 5% normal donkey or goat serum in PBS) for 1 h at room temperature (20–23 °C). Next, the sections were incubated overnight at 4 °C in blocking–permeabilizing solution containing mixes of the following antibodies: rabbit anti-MAP2 (1:1,000; Sigma-Aldrich, AB5622), mouse anti-OTX2 (1:200; Invitrogen, MA5-15854), goat anti-FOXA2 (1:200; R&D systems, AF2400), mouse anti-TH (1:70; Novus Biologicals, MAB7566), chicken anti-CALB1 (1:1,000; Novus Biologicals, NBP2-50028SS), guinea pig anti-VGLUT1 (1:250; Merck, AB5905), rabbit anti-LHX9 (1:30; Merck, HPA009695), chicken anti-Doublecortin (1:1,000; Abcam, ab153668), mouse anti-PAX2 (1:500; Antibodies.com, A252739), goat anti-ZIC1 (1:40; Thermo Fisher Scientific, PA5-47681) and rabbit anti-GlyT2 (1:500; Thermo Fisher Scientific, MA5-52662).

The next day, the sections were rinsed five times in PBS before incubation for 2 h at room temperature with 1:300 secondary antibody (donkey: anti-rabbit AF 488, Invitrogen, A32790; anti-rabbit AF 568, Invitrogen, A10042; anti-rabbit AF 647, Invitrogen, A31573; anti-mouse AF 568, Invitrogen, A10037; anti-chicken AF 488, Invitrogen, A78948; anti-goat AF 488, Invitrogen, A11055; anti-goat AF 647, Invitrogen, A21447; anti-guinea pig AF 488, Jackson Immunoresearch (Lucerna Chem) 706-545-148. Goat: anti-rabbit AF 488, Invitrogen, A11034; anti-mouse AF 568, Invitrogen, A11031; anti-chicken AF 647, Invitrogen, A32933; anti-guinea pig AF 568, Invitrogen, A11075) in blocking–permeabilizing solution with DAPI at 0.2 µg ml^−1^. Finally, the secondary antibody solution was washed off with PBS before covering with ProLong Gold Antifade Mountant medium (Thermo Fisher Scientific). Stained organoid cryosections were imaged using a Nikon W1-SoRa Inverted Spinning Disk confocal microscope, and six different z-plane images (z-step, 2–3 μm) were acquired using a ×20 (day 180 organoids) or ×40 (day 30 organoids) magnification objective. The images were further processed using the NIS-Elements software and Fiji (ImageJ).

#### Spatial transcriptomics experiment

To design the 500-gene panel for the spatial transcriptomic measurement with MERSCOPE^51^, we combined a pre-defined gene list, which includes canonical regional and cell type markers, and an additional list of genes selected by geneBasis^52^ (Supplementary Table 10).

We curated two datasets for the gene panel selection. The first included public scRNA-seq datasets of brain organoids cultured for no more than 1 month^27,31,53–55^, together with the scRNA-seq portion of the time-course scMultiome data from days 7, 15 and 30 presented in this study (the early dataset). All included scRNA-seq data were merged, normalized and scaled (for the 3,000 highly variable genes). Principal component analysis (PCA) was applied to the scaled data for the first 20 principal components (PCs). CSS^56^ was applied for data integration on samples. Louvain clustering (resolution = 2) was performed to identify cell clusters for early brain organoid cultures. In addition, a merged dataset of two public scRNA-seq data sets^53,54^ of brain organoids older than 1 month (the late dataset) was curated and processed (union of highly variable genes per dataset and the merged dataset; PCA on the scaled data for the top 20 PCs; CSS integration on samples; Louvain clustering with resolution of 3 on the CSS representation). Differentially expressed genes (DEGs) were identified for each cluster with the wilcoxauc() function in the presto package (adjusted *P* < 0.01, fold change of >1.2, area under the curve (AUC) of >0.65, detection rate difference of >20% and detection rate ratio of >2; maximum 20 genes per cluster based on the AUC value). The DEGs were merged and examined in cluster pairs along the branch merging on the dendrogram of clusters based on their expression distances. Clusters with no more than five overall DEGs showing differential expression were merged, resulting in the final clustering results for the late dataset. Average gene expression profiles were estimated for each cluster in the early and late datasets in units of transcripts per million (TPM). For the pre-defined gene list, genes are required to satisfy one of the following criteria: (1) maximal average expression in the early dataset clusters between 1 and 500 TPM; (2) maximal average expression between 500 and 1,000 TPM but not clustered with any genes selected by criteria 1; or (3) maximal average expression in the late dataset clusters between 1 and 500 TPM. This resulted in a total of 242 genes.

Next, we used geneBasis to complete the 500-gene panel, using the early dataset. To reduce computational complexity, we reconstructed metacells for each sample separately using the previously described approach^22,53^, applying global PCs for neighbor graph construction and a downscaling ratio of 1:10. This approach pooled gene counts from transcriptomically similar cells from the same samples, followed by normalization to total transcript counts per metacell. The normalized expression of metacells was then provided to geneBasis to target 600 genes in total, given the pre-selected genes mentioned above and a gene list to drop from the selection (the union of genes with maximal average expression in the early dataset cluster of <1 TPM and those with >500 TPM, cell-cycle-related genes, mitochondrial genes and ribosomal protein genes). The resulting gene list was further filtered by the Vizgen database, and the top 500 genes remaining were chosen for the spatial transcriptomics experiment by MERSCOPE.

Two organoids based on protocol 1 (ref. ^57^) at day 30 of their culture were fixed in 4% PFA for 1.5 h and subsequently exposed to sucrose gradients (15% and 30%) until the organoids sank. Organoids were then co-embedded and frozen in an OCT block (Tissue-Tek OCT Compound, Sakura) before being stored at −80 °C. To increase the adherence of organoid slices to the MERSCOPE slides (Vizgen, 10500001), the slides were coated with 1 ml of poly-D-lysine (Thermo Scientific, A3890401) for 3 h and washed with 2 ml of RNAase-free water three times. The slides were then dried and left at room temperature. The block was incubated for 30 min at −20 °C in a cryostat (ThermoFisher, CryoStar NX50 Cryostat), and 10 µm-thick sections were cut. Multiple slices representing different z-planes of the organoids were mounted on two warm, functionalized, bead-coated MERSCOPE slides. The slides were then placed in a 60 mm Petri dish and stored in the cryostat for 20 min, then dried at 50 °C for 30 min before washing with 5 ml 1× PBS (Invitrogen, AM9625) three times for 5 min each at room temperature to remove the OCT. Then, 5 ml of 70% ethanol was added to the Petri dish for permeabilization. Samples were photobleached for 3 h to reduce the tissue background during imaging using the Vizgen Photobleacher (Vizgen, 10100003). The samples were then washed with 5 ml Sample Preparation Wash Buffer (Vizgen, 20500001-2) before adding 5 ml Formamide Wash Buffer (Vizgen, 20500001-2) for 30 min at 37 °C and then hybridized with the MERSCOPE Gene Panel Mix at 37 °C in an incubator for 42 h. Next, the tissue slices were washed twice with 5 ml formamide wash buffer at 47 °C for 30 min and embedded into a hydrogel using the Gel Embedding Premix (Vizgen, 20500001-2), ammonium persulfate (Sigma-Aldrich, A3678-25G) and TEMED (*N*,*N*,*N*′,*N*′-tetramethylethylenediamine) (Sigma-Aldrich, T7024-25ML) from the MERSCOPE Sample Prep Kit (Vizgen, 20500001-2). After 1.5 h, the gel embedding solution polymerized, and the sample was cleared with a clearing solution consisting of 50 µl of Proteinase K (NEB, P8107S) and 5 ml of Clearing Premix (Vizgen, 20500001-2) at 37 °C overnight. Then, the samples were washed with 5 ml sample preparation wash buffer and imaged on the MERSCOPE system (Vizgen, 10000001) using a MERSCOPE 500-gene imaging kit (Vizgen, 10400012). A step-by-step instruction on the MERFISH sample prep is available at https://vizgen.com/resources/fresh-and-fixed-frozen-tissue-sample-preparation, and the instrumentation protocol is available at https://vizgen.com/resources/merscope-instrument.

#### Generation of a doxycycline-inducible Cas9 nuclease stable cell line

The generation of a doxycycline-inducible Cas9 nuclease stem cell line was achieved through the stable integration of inducible Cas9 and reverse tetracycline-controlled transactivator (rtTA) into the AAVS1 safe-harbor locus using transcription activator-like effector nucleases (TALEN). To construct the donor vector containing Cas9 with dual nuclear localization signals (dual-NLS), the SV40-NLS and nucleoplasmin-NLS sequences were appended to the 5′ and 3′ ends of Cas9 (from the Puro-Cas9 donor; Addgene, 392399) by PCR amplification. Before PCR, the Puro-Cas9 donor template was digested with AgeI and AscI to minimize background during cloning. The amplified dual-NLS Cas9 fragment was inserted into an AAVS1 Hygro-donor vector (Addgene, 86883), downstream of a tetracycline-responsive element promoter, using NEBuilder HiFi DNA Assembly (NEB, E2621L) following the manufacturer’s instructions. The generation of the doxycycline-inducible Cas9 nuclease (iCas9) stem cell line was performed in accordance with previously published protocols^58,59^ with minor modifications^36^. In brief, four plasmids, including dual-NLS-iCas9-Hygro, AAVS1-Neo-M2rtTA (Addgene, 60843), AAVS1-TALEN-L (Addgene, 59025) and AAVS1-TALEN-R (Addgene, 59026), were prepared using endotoxin-free maxiprep kits (Qiagen, 12362) and nucleofected into WTC iPS cells. A total of 20 µg DNA with an 8:8:1:1 ratio was nucleofected using the Lonza 4D-Nucleofector X following the manufacturer’s protocol. Post nucleofection, the WTC cells were plated on a 10 cm dish pre-coated with Matrigel in mTeSR+ medium supplemented with 5% CloneR (STEMCELL Technologies, 05888). G418 selection (100 µg ml^−1^) was performed from days 2–5, followed by hygromycin selection (50 µg ml^−1^) from days 7–9. Single clones were subsequently picked and screened for dual insertion of Cas9 and rtTA through genomic DNA PCR, followed by qPCR to assess doxycycline-inducible Cas9 expression^58,59^.

#### Cloning and lentivirus packaging for the perturbation experiment

TF-KO gRNAs were designed using CHOPCHOP and Synthego. To assemble vectors for each TF-KO, gRNA cassettes were assembled from PCR primers (IDT; Supplementary Table 6), amplified and inserted into a template vector. Each lentivirus carried two guides, targeting the same gene. Gel-purified cassettes were assembled into a linearized vector using Gibson assembly (NEB, E2621). The presence of the correct dual gRNA cassettes was verified by Sanger sequencing. Lentivirus was produced in-house following a four-plasmid second-generation lentivirus protocol requiring TAT-proteins for LTR-dependent integration into the genome. A 1:1:1:1:4 plasmid mix consisting of 1.5 μg of each Gag-pol, Vsvg, Rev and Tat helper plasmids with 6 μg of the respective lentivirus vector was used for transfection into 80–90% confluent HEK293T cells using TransIT-293 reagent (Mirus Bio, MIR 2705). Cells were cultured in high-glucose DMEM (Gibco, 41965039) supplemented with 10% (v/v) FBS (Sigma-Aldrich, F2442), 1× GlutaMAX (Gibco, 35050061) and 0.2% (v/v) penicillin–streptomycin (Gibco, 15140122). To purify lentivirus, the medium was changed to 30% FBS 1 day after transfection and collected after 24 h. A 50× lentiviral suspension was generated in DPBS using Lenti-X concentrator (Takara Bio, 631232). Lentivirus was produced separately for each KO construct.

#### Stable cell line and mosaic organoids generation for perturbation experiment

A clonal doxycycline-inducible Cas9 iPSC line with WTC background (Coriell Institute), generated following previously established protocols^36^, was infected in a pooled fashion (Supplementary Table 6). As a control, lentivirus harboring non-targeting guide sequences was used. Cells were cultured for 8 days after infection under continuous selection with 2 μg ml^−1^ blasticidin (Gibco, R21001). After selection and visual confirmation of fluorescent marker expression, cells were dissociated using TrypLE (Gibco, 12605010) and underwent further selection for positive fluorescence using FACS. Selected cells were plated and allowed to recover for 2–3 days (80–90% confluency) before being cryopreserved.

Cryopreserved cell pools were thawed and passaged once before cell suspensions were generated using TrypLE (Gibco, 12605010). A final cell suspension was created by mixing the KO pools (Supplementary Table 6) to ensure each KO was represented equally in the final cell suspension. The final mixed pool of cells was used to generate Qian organoids, following the previously described method^14^. To induce Cas9 expression and therefore create KOs in targeted genes, 2 μg ml^−1^ doxycycline (Clontech, 631311) was added with each media change starting on day 7 until day 14. Organoids were returned to their normal media on day 15. Fluorescence was monitored throughout culturing to ensure KO reporters were not silenced throughout differentiation. Five organoids were pooled and dissociated into single-cell suspensions at day 30 and day 70. Cells positive for fluorescent reporters were selected using FACS, and these cells were then loaded onto the 10x Genomics Chromium controller using the Chromium Next GEM Single Cell 5′ v2 Dual Index kit (10x Genomics, PN-1000265), targeting 10,000 cells per reaction with two reactions per time point. Single-cell transcriptome and CRISPR gRNA libraries were prepared following manufacturer protocols. The transcriptome libraries and gRNA libraries were pooled and sequenced on the NovaSeq 6000 platform using 26/10/10/90 as cycle parameters.

#### Cutting efficiency validation for the perturbation experiment

A clonal doxycycline-inducible Cas9 iPS cell line (WTC background, Coriell Institute), generated following previously established protocols^36^, was infected in an arrayed fashion. After culturing with virus for 2 days, cells were cultured under continuous blasticidin selection conditions for 6 days. After selection and visual confirmation of fluorescent marker expression, Cas9 expression was induced with 2 μg ml^−1^ doxycycline for 48 h. After that, total DNA was extracted using the QuickExtract DNA kit, and corresponding DNA regions targeted by gRNA were amplified, followed by indexing PCR with Nextera XT Index Kit v2 Set B primers. All samples were pooled together and sequenced with the MiSeq V2 Nano platform using 150/8/8/150 as cycle parameters. Efficiency was estimated using CRISPResso2 (ref. ^60^).

#### Organoid culture for the morphogen screen experiment

Brain organoids were generated from the WTC cell line. For the morphogen screen experiment, protocol 1 (ref. ^14^) was used as a baseline and also serves as a control. Organoids were plated in ultra-low-attachment plates (Corning, CLS7007), with five organoids per condition. On day 15, they were transferred into six-well plates, placing all five organoids per condition in one well, and kept on a shaker, as in the original protocol. The majority of morphogens were provided between days 8 and 14 (Supplementary Table 5), always using base medium as indicated in the original protocol. An exception was SHH, which was provided from days 1–7, and CHIR, from days 5–14, as in the original protocol; insulin, which was provided from days 1–14 as in the published cerebellum protocol^19^; and FGF19, which was provided from day 15 onwards, as it has been known to promote neural differentiation. We used the following morphogens: SHH (R&D Systems, 1845-SH-025/CF), CHIR-99021 (Tocirs, 4423), insulin (Sigma-Aldrich, I9278), R-Spondin-3 (Peprotech, 120-44), R-Spondin-2 (Peprotech, 120-43), retinoic acid (Sigma-Aldrich, R2625), FGF8 (STEMCELL Technologies, 78204), FGF19 (Peprotech, 100-32), FGF17 (Peprotech, 100-27), FGF2 (Mitenyli Biotech, 130093840) and BMP4 (Mitenyli Biotech, 130-111-167).

#### Single-nuclei isolation, fixation, snRNA-seq library preparation and sequencing for the morphogen screen experiment

After 5 weeks (36 days) in culture, one to four organoids per condition were dissociated individually using the CyBio FeliX liquid handler robot with a thermoshaker. For dissociation, we used a papain-based dissociation kit, as with the time course. Each organoid was dissociated using 820 μl of enzyme mix 1 and 12 μl of enzyme mix 2. Then, each individual single-cell suspension underwent nuclei isolation, as described for the time course (lysis time, 2.5 min). Nuclei fixation and permeabilization procedures were performed according to the manufacturer’s specification (ParseBiosciences, Nuclei Fixation kit v2.1.2, WN400). Then, the collected samples were processed for highly multiplexed snRNA-seq using a split-pool combinatorial barcoding kit (ParseBiosciences, WT Mega kit v2, dual-index version RX200).

### Data analysis methods

#### Preprocessing of scMultiome data from the organoid time course

We used Cell Ranger ARC (v2.0.0) with the default parameters to map the RNA-seq and ATAC-seq portions of the data to the human reference genome and gene annotation provided by 10x Genomics (GRCh38-2020-A-2.0.0). All samples were aggregated using the aggr command in Cell Ranger ARC to have the same list of accessible peaks quantified for all cells. Both data modalities were read and further processed in R using Seurat (v4.4.0)^61^ and Signac (v1.1.1). As quality control, only cells meeting the following criteria were retained: detected gene number between 1,000 and 7,500; percentage of mitochondrial transcripts less than 30%; detected ATAC-seq fragment number between 1,000 and 30,000; nucleosome signal less than 2.5; and transcription start site enrichment higher than 1.

Nuclei from different stem cell lines were demultiplexed using the demuxlet^62^ tool. Genotyping information was downloaded from the HipSci (WIBJ2, HOIK1) or Allen Institute (WTC) website. Bcftools were used to merge all vcf files, and sites with the same genotypes in all samples were filtered out. Demuxlet was run with default settings on transcriptomic and genomic reads. Cells with ambiguous assignments were classified as ‘unknown’. For all other cells, the best singlet assignment was considered.

To analyze the scRNA-seq modality, the standard log-normalization was first applied, and the top 3,000 highly variable genes were identified. Subsequently, truncated PCA was performed with the scaled expression levels (across all cells) of highly variable genes as the input, using the RunPCA() function from the Seurat package. The first 20 PCs were used to integrate different samples in the dataset (time point and protocol) using the CSS method^56^ (cluster resolution = 1.2). We performed uniform manifold approximation and projection (UMAP)^63^ to obtain a two-dimensional representation of the data. We used the RunUMAP() function with default parameters using all components of the CSS matrix.

For the scATAC-seq modality, the tf-idf (term frequency times inverse document frequency) normalization, implemented as the default normalization method in the Signac package for scATAC-seq data, was first applied. Singular value decomposition was then performed using the RunSVD() function from the Signac package on the normalized counts of the top peaks. The first 50 latent semantic indexes, except for the first one because of its high correlation with numbers of fragments per cell, were used for scATAC-seq data integration using the CSS method (cluster_resolution = 0.8), followed by performing UMAP for low-dimensional data representation.

#### Cell type annotation of the scMultiome data

To annotate the data, we first identified clusters using the RNA assay, based on the CSS integrated embeddings, with the FindNeighbors() (default parameters) and FindCluster() (resolution = 0.5) methods implemented in Seurat. Clusters were annotated into cell classes (NPC, neuroblast, neurons, astrocytes, neural crest derivatives, mesenchymal cells) based on canonical marker expression. Neuron clusters were further annotated based on neurotransmitter transporter expression into glutamatergic neurons, GABAergic neurons (not Purkinje cells), Purkinje cells, dopaminergic neurons and glycinergic neurons.

To further disentangle heterogeneity of dopaminergic neurons, we subsetted 4,171 cells that were classified as dopaminergic neurons and re-preprocessed scRNA-seq data: normalization, highly variable genes selection, scaling and integration. For integration with the CSS method, the first 20 PCs were used, with a cluster resolution of 1.2. UMAP embeddings were obtained using the CSS matrix. Louvain clustering (resolution = 0.5) was applied to identify subtypes of dopaminergic neurons. For dopaminergic neuron subtype classification, we used canonical markers from the literature^64–67^. We observed strong expressions of *FOXA2*, *LMX1A*, *NR4A2* and *EN1*, which indicate midbrain origin of dopaminergic neurons^7^. We detected subtypes that resemble ones previously described^65^ (hDA1); however, we did not observe expression of *ALDH1A1* and *LMO3*, which would be specific for hDA2.

To further dissect the heterogeneity of glutamatergic neurons, we subsetted the 7,372 glutamatergic neurons that we annotated. For the scRNA-seq portion, 3,000 highly variable genes were re-identified. Their scaled expression levels across cells were used to rerun the PCA, with the first 20 PCs being used to run CSS integration across samples. To incorporate the scATAC-seq information, the weighted nearest neighbor graph approach, implemented as the FindMultiModalNeighbors() method in Seurat, was applied to estimate modal weights for each cell in the subset, resulting in a nearest neighbor graph incorporating both the scRNA-seq and scATAC-seq information. Louvain clustering (resolution = 1) was then used to identify clusters, which were annotated based on marker expression and the mapping to the primary reference.

#### Mapping of scRNA-seq data to primary reference

We adapted the projection and comparison strategy as previously described^22^ to compare the transcriptomic portion of the scMultiome time-course data to the human first-trimester developing brain cell atlas^28^. In brief, the scANVI model and the primary atlas data were retrieved. The scRNA-seq portion of the time-course data was projected to the primary scANVI latent space using scArches^68^. A bipartite weighted *k*-nearest neighbor (wkNN; *k* = 50, weighting_scheme, ‘jaccard_square’) graph was constructed. Based on the reconstructed wkNN graph, cell class label transfer was done as a weighted majority voting. The regional labels were transferred to each cell in the time-course data in a similar manner, but hierarchically, to take into account the brain region hierarchy, as described previously^22^.

Based on the reconstructed wkNN graph, for a given subset of the query data (for example, all cells of one sample or cell type), a presence score can be computed for each cell in the reference atlas as the sum of edge weights on the wkNN graph, as a metric of the likelihood that the cell type or state represented by the reference cell is present in the given query cell population. To summarize the presence scores of multiple query populations, the presence scores of each query population were min–max normalized across all the reference cells, followed by a max-pool across all query cell populations.

To estimate transcriptomic similarity between cells in the organoids and human developing brains, we adapted the matched metacell reconstruction strategy as described previously^22^. In brief, for each query cell (that is, every cell in the time-course data), the average expression of its neighbors in the reference was calculated, weighted by the weights of the wkNN graph. Transcriptomic similarity was then quantified per cell as the Pearson correlation between a query cell and its matched metacell across highly variable genes. To compare expression profiles of organoid cells and their matched primary metacells, an *F*-test-based paired differential expression analysis proposed previously^22^ was used, which estimates statistical significance of whether a gene’s expression difference between query cells and their matched metacell is systematically different from zero.

To compare to the second-trimester developing human brains, we collected scRNA-seq data of eight public scRNA-seq atlases with 2nd-trimester developing human central nervous system (CNS; brains and spinal cord) samples^28,69–75^, targeting single or multiple brain regions. An automatic quality control procedure, as reported previously^22,53^, was applied to each sample, resulting in 1.5 million cells in total. The data predominantly represent cerebral cortex (530,000, 35%) and spinal cord (566,000, 37%). The non-cortical brain data are mostly thalamus (162,000, 11%) and cerebellum (89,000, 6%). Author-provided cell class annotations were summarized into ten categories (NPC, neuron, glioblast, astrocyte, oligonucleotide, Schwann, choroid plexus, immune, endothelial, mesenchymal). WkNN-based label transfer^22^ was used to infer cell class information for 618,000 cells without annotation based on the PCA embedding. In addition to the dissection-based region information, region information was also transferred from the integrated developing human CNS atlas, focusing on the first-trimester samples. AggreCells^22,53^ were generated from the data with 10% sampling rates per sample. Data integration was performed using scVI on aggreCells with donors as batches. The resulting model was used to initialize the label-aware scANVI model inference, given the region + cell-class labels as the cell type labels. After the model was trained using aggreCells, it was applied to the single-cell data to obtain the single-cell latent representation. Next, scArches was applied to the scRNA-seq portion of the time-course data. A similar analysis as described above was applied to compare the transcriptomic portion of the time-course data to the human second-trimester developing CNS cell atlas.

#### Spatial transcriptomic data preprocessing and analysis

The raw images of DAPI and Poly-T were retrieved from the MERSCOPE data output folder. For each tissue section, organoid masks were created by binarizing the DAPI image (*z* = 0), filling the holes and identifying the connected pixels. For each organoid mask, the corresponding DAPI and Poly-T images were then cropped into its expanded bounding box (both *x* and *y* axes expanded 500 pixels on each side).

For each cropped DAPI and Poly-T image, an intensity normalization was then applied, for each z-stack separately. In brief, for each image, the local minimum (denoted as *x*_0_) between the two peaks of its intensity distribution was first identified based on binning as one normalization anchor. The other normalization anchor (*x*_1_) was defined as the 99.9^th^ percentile of the image intensities. The normalized intensity was then calculated as *x*′ = (*x* − *x*_0_) / (*x*_1_ − *x*_0_). Next, cellpose (v2.0)^76^ was applied for segmentation, using the cyto2 model on the Poly-T and DAPI channels. The segmentation was first done on each z-stack separately. The segmented masks were then stitched and harmonized across z-stacks using the stitch3D function in cellpose (Extended Data Fig. 3). Cell masks with fewer than 3,000 pixels were excluded. Furthermore, any scatter cell mask, defined as those with fewer than seven cell masks within a distance of 250 pixels, was excluded.

Next, for each segmented cell after the filtering, transcripts being called by the MERSCOPE analytic pipeline and overlapping with the cell mask were counted for each gene to quantify its gene expression profile. Cells with fewer than 40 detected transcripts were excluded. The transcript numbers per cell (*n*) were then normalized to the cell volumes (*v*, number of pixels) with a target sum of 40,000: *n*′ = *n* / *v* × 40,000.

To generate the UMAP of the MERSCOPE data, the volume-normalized expression levels of each of the 500 measured genes were log transformed (with one pseudocount, using the pp.log1p function in scanpy) and scaled (pp.scale function in scanpy). PCA was applied to the scaled expression matrix. The first PC was discarded for its high correlation with the number of detected transcripts per cell. The second to eleventh PCs were used to generate the neighbor graph using the pp.neighbors function in the scanpy package, followed by generating the UMAP embedding with the tl.umap function in the same package.

To transfer the cell type labels from the scMultiome data, we first generated a subset of the scMultiome data with matched time point and organoid culture protocol as the MERSCOPE data, by selecting cells from samples representing day 30 organoids generated with protocol 1. Cells with cell type labels of cell types with no more than 100 cells in the subset were further excluded from the subset. Next, we generated the UMAP embedding of the subset based on its CSS representation calculated earlier (default parameters) and performed Louvain clustering at high resolution (resolution = 5) based on its neighbor graph (default parameters). For each of the high-resolution clusters, the average expression of the 500 genes measured in the MERSCOPE experiment was calculated, and the cell type label with the highest frequency among cells in the cluster was assigned to the cluster. Next, the expression profile of the 500 genes for each cell in the MERSCOPE data was correlated with each of the high-resolution clusters. The cell type label of the most correlated cluster was assigned to the MERSCOPE cell.

#### Characterization of chromatin accessible regions with cisTopics and differential accessibility analysis

We combined the cisTopic analysis and differential accessibility analysis to characterize the detected accessible regions in the scATAC-seq portion of the scMultiome data of the time-course atlas. To run cisTopic^29^, we used create_cistopic_object_from_fragments() in the pycisTopic package to generate a cell-count matrix from fragments. Considering the large size of the dataset, we ran this analysis on the subsetted dataset (1,000 cells per cell type). We performed topic modeling, varying the number of topics from 50 to 300, and selected the model with 250 topics. The estimated topic loadings were considered as representations of the detected accessible regions. PCA was thus applied to the topic loadings, followed by Louvain clustering (resolution = 1) to identify region clusters based on the neighbor graph of accessible regions estimated with the first 50 PCs. To visualize clusters of the chromatin regions, *t*-distributed stochastic neighbor embedding was calculated (with default parameters). All the above analysis based on the cisTopic output was done with the scanpy package in Python (Supplementary Tables 3 and 4).

We also adapted a differential accessibility analysis^53^ to estimate the statistical significance of accessibility differences among different cell types. In brief, the chromatin accessibility count matrix was first binarized. For each accessible region, two generalized linear models with quasibinomial error distribution were fit. The full model includes two independent variables: the number of detected fragments per cell and the cell type label; the reduced model includes only the number of detected fragments per cell as the independent variable. The variation residuals of the two models were then compared using an *F*-test. The resulting *P* values were adjusted using the Bonferroni method. Accessible regions with adjusted *P* values of <0.1 were considered differentially accessible across cell types. Fisher’s exact tests were used to check the enrichment of differentially accessible regions in each region cluster.

#### Characterization of chromatin accessible regions clusters with GREAT and HOMER

We used the rGREAT package^77^ and the Gene Ontology biological processes database to perform pathway enrichment analysis of the peak clusters. For each cluster, the top ten terms (based on the lowest *P* value adjusted using a hypergeometric test over genes) were selected for presentation.

We used HOMER software^78^ and the JASPAR 2024 database^79^, a subset of vertebrate motifs, to perform motif enrichment analysis of peak clusters. For each cluster, the top ten terms (based on the lowest *P* value) were selected for presentation. For both analyses, only peaks classified as differentially accessible (see previous section) were considered.

#### Mapping scATAC-seq data to the primary reference using developed ATAC-mapper package

First, we generated a cell-count matrix from fragments for the organoid atlas, using peak ranges defined in the human developing brain chromatin accessibility cell atlas^30^ and the FeatureMatrix() function from the Signac R package. Next, for each cell, we inferred the topic distribution using peak count information and topic-peak loadings derived from the cisTopic model presented in the primary cell atlas. Inference of topic profiles for query cells was done with an expectation–maximization-like process. Each iteration includes: (1) computing the posterior probability of each topic given each peak by multiplying topic-peak loadings with the topic distribution vector from the previous iteration; (2) normalization across topics to ensure that probabilities are summed to one for each peak; (3) refining the overall topic distribution using peak probabilities by multiplying the term frequency vector (peak counts vector) with the posterior probabilities of each topic given each peak; and (4) re-normalization of topic probabilities. For each cell, a maximum of 100 iterations was applied to estimate the topic probabilities. Alternatively, if the difference between an updated topic distribution and the previous one was less than 1 × 10^−4^, the probability inference was considered to be converged, and the inference ended. The explained algorithm is adapted from the Python package lda^80^.

Next, to map the scATAC-seq data to the primary reference based on the inferred topic profiles, we applied scPoli to the primary scATAC-seq reference data, given the topic profiles (scaled across cells) as the input, with the donor information as the batch covariate, and the following information as the cell type labels: Cellclass and Celltype. The batch covariate was represented in the model as a learned vector of size five. We chose the hidden layer size of the one-layer scPoli encoder and decoder as 128, and the latent embedding dimension as 20. We trained the model for a total of 50 epochs, 35 of which were pre-training epochs. With the trained scPoli model, we used scArches to map the scATAC-seq portion of the scMultiome time-course atlas to the reference scPoli latent space. The inferred topic profiles were first scaled with the same scaling factors as the reference data. Next, the query model was fine-tuned with a batch size of 2,048 for a maximum of 20 epochs.

With the reference data and the query data represented with the same latent representations, we adapted the bipartite wkNN graph-based comparison for label transfer, presence score estimation and matched metacell reconstruction (on topic profile level) described above to compare the scATAC-seq portion of the scMultiome time-course organoid cell atlas and the human developing brain chromatin accessibility cell atlas.

The package for mapping ATAC data to primary atlases is available on GitHub https://github.com/quadbio/atac_mapper and PyPi https://pypi.org/project/atac-mapper. The algorithm provides the mapping result on a single-cell level. The approach identifies the nearest neighbors of query cells across reference atlases and is cluster-agnostic.

#### Estimating cell line and cell type specificity for open chromatin peaks

In order to estimate the connection between opened chromatin peaks and cell lines or cell types, we used the following approach. Peak counts in cells were binarized (if a peak had more than one count in the cell, the peak was considered present). Then the mutual information between peak and assigned cell lines, conditioned on cell type, was calculated, taking into account all cells of the dataset. The reverse procedure was also done, calculating mutual information between peak and cell types, conditioned on the cell line.

#### Calculation of motif enrichment scores for single-cell atlas

Transcription factor binding motifs (TFBSs), the sequence motifs that different TFs recognize and bind, were retrieved from the JASPAR 2024 database^79^ using the JASPAR2024 R package in the format of position frequency matrices. The TFBS information was added to the scMultiome time-course atlas data with the AddMotifs() function in Signac. The chromVAR analysis^81^ was then run using the RunChromVAR() wrapper function in Signac (default parameters), to estimate the enrichment of each TFBS at each cell in the atlas.

#### GRN inference with Pando

First, we excluded non-neuronal cell types (mesenchyme and neural crest) and non-CNS cell types (spinal cord neuroblasts). GRN initiation and motif search were performed using default parameters of the Pando framework (v1.0.0). GRN inference was performed using highly variable genes and ATAC peaks aggregated at the high-resolution cluster level to reduce noise in the data. We used a regularized linear model (cv.glmnet) with the elastic net mixing parameter 0.5. Modules were defined using find_modules_network() function with the following parameters: rsq_thresh = 0.1; nvar_thresh = 10; min_genes_per_module = 10. Module activity was calculated using the Seurat function AddModuleScore(), based on target genes inferred by Pando.

#### Estimating regional and cell type specificity of regulons for NPCs and neurons

To characterize the differences in TF usage of NPCs with midbrain and hindbrain identities, we extracted the subset of NPCs with midbrain and hindbrain regional annotation from the time-course data. Focusing on the 246 TFs that were detected in the data, with TFBS motif information in JASPAR 2024 and regulon inferred by Pando, Wilcoxon’s rank-sum test, implemented as the wilcoxauc() function in the presto package in R, was applied to compare the gene expressions, corresponding TFBS enrichments (the chromVar estimated scores) and regulon activities between midbrain NPCs and hindbrain NPCs. TFs with significant differences (expression: adjusted *P* < 0.01, fold change of >1.2, AUC > 0.6, detection rate difference of >10%; chromVar score and regulon activity: adjusted *P* < 0.01, AUC > 0.7) were considered as showing regional differences in NPCs and potentially responsible for regional identity establishment.

To characterize differences in TF usage across different neuron cell types, we first extracted the subset of neurons from the time-course data. To identify TFs representing distinct neuron cell types based on expression, motif enrichment and regulon activity levels, we performed two different statistical tests on each level separately. Similar to the analysis on NPCs, we applied the Wilcoxon rank-sum test implemented in the presto package as the first test to identify TFs with differences among cell types on different molecular levels (expression: adjusted *P* < 0.01, fold change of >1.2, AUC > 0.6, detection rate difference of >10%; chromVar score and regulon activity: adjusted *P* < 0.01, AUC > 0.7). In addition, we implemented an *F*-test-based differential abundance test. The test compares the residuals of two linear models: the full model with both cell types and a neuron–NPC score as described previously^56^ as a covariate, and the reduced model with only the neuron–NPC score. The neuron–NPC score is calculated as the difference in the module scores (with the AddModuleScores() function in Seurat) of neuron-high genes and those of NPC-high genes, and therefore represents the progress of neural differentiation. With the second test, any comparison with Benjemini–Hochberg-adjusted *P* < 0.05 was considered statistically significant. For each of the molecular levels, a TF was considered differentially associated across neuron cell types if both tests indicated significant differences. Next, a TF was considered to contribute to the differences among different neuron cell types if its regulon showed differential activities, plus significant differences detected in either gene expression or motif enrichment level.

To compare regional differences of TF profiles in NPCs and neurons, we curated two lists of TFs with high confidence of positive regulation of their targets. For NPCs, we required the differences between average levels in midbrain and hindbrain having the same sign for TF expression, the enrichment of their corresponding TFBS motifs and their positive regulon activities. For neurons, we first calculated Pearson’s correlation of TF expression and positive regulon activity with motif enrichment for each TF across neuron cell types. TFs with at least one correlation of >0.5 and neither being <0.1 were considered to be high-confident positive regulators in neurons. The two lists were unioned, and the differences between their average levels in midbrain and hindbrain were calculated for NPCs and neurons separately. The profiles of the NPC and neuron regional differences were then correlated for each of TF expressions, TFBS motif enrichments and positive regulon activities. Feature (TF/motif/regulon) label permutations were performed 100 times, separately for TF expression, TFBS motif enrichments and positive regulon activities, to estimate the corresponding null background and determine statistical significance.

To generate a low-dimensional representation of TF specificity across neuron cell types in the organoids, we focused on high-confidence positive TFs in neurons and applied PCA to the scaled average regulon activities in different neuron cell types. The first and fifth PCs were selected as the 2D embeddings of TF neuron cell type specificity, with which TFs with the highest regulon activity at the same cell types tend to be separated. The cell type and regional specificity of TFs were also examined by comparing regulon activities and TFBS motif enrichment, with the assumption that TFs responsible for maintaining cell type identity of a neuron cell type should be high in both values.

#### Processing and analysis of CRISPR-perturbation scRNA-seq data

We used Cell Ranger (v7.0.1) using flag multi to obtain count matrices of transcriptome and gRNAs. We used the human transcriptome (hg38, provided by 10x Genomics) and a table of guide sequences for GEX and gRNA reads mapping, respectively. To facilitate efficient gRNA detection, the pattern and sequence entries in the feature reference files were adjusted to 12 bp instead of 20 bp. Then, as with the time course, count matrices were further processed using the Seurat R package. Cells were filtered based on the number of counts (>600, <20,000 for day 30) or detected genes (>500, <7,500 for day 70) and the fraction of mitochondrial genes (<0.15 for day 30 and <0.1 for day 70). Transcript counts were normalized to the total number of counts for that cell, multiplied by a scaling factor of 10,000 and subsequently natural-log transformed (NormalizeData()). The 10X lanes from the same time points were integrated using the CSS method as for the time-course data. Datasets were annotated by combining label transfer from the time-course dataset (using the css_project() function of the CSS package) and marker gene expression.

To assign gRNA labels, the 10x gRNA calling outputs (protospacer_calls_per_cell.csv) were first filtered to retain cells with gRNAs targeting a single gene. For cells with gRNAs targeting multiple genes, additional filtering was applied to identify the best call, requiring a z-scaled unique molecular identifier (UMI) value of >5 and a total UMI count of >10. Cells lacking gRNA calls were excluded from further analysis. Cells with effective perturbations were identified using Mixscape^82^ as implemented in Seurat, using cells with all other gRNAs as a control. Regulon activities upon CRISPR-based KO were quantified using the Seurat function AddModuleScore(), considering the positive and negative regulons of the TF of interest as inferred in Pando. To visualize the detected gRNAs and regulon activities on the UMAP embeddings, their densities were estimated using Nebulosa^83^ (weight = 1 for gRNA). DEGs were identified for each cluster with the FindMarkers() function in the Seurat package using a Wilcoxon test (log_2_ fold change of >0.5, adjusted *P* < 0.05, detection rate of >5%, detection rate difference of >10%) and analyzed for Gene Ontology enrichment using clusterProfiler^84^.

#### Processing and analysis of morphogen perturbation screen scRNA-seq data

We used Parse Biosciences Software (v1.0.4) to demultiplex barcodes, map to the hg38 human transcriptome and generate a count matrix, which was further processed using the Python scanpy package (v1.10.3)^85^. Cells were filtered based on UMI counts (>1,000, <30,000) and the fraction of mitochondrial genes (<10). Transcript counts were then normalized to the total number of counts for that cell, multiplied by a scaling factor of 10,000 and subsequently natural-log transformed. Highly variable genes were estimated, and total UMI counts and the fraction of mitochondrial genes were regressed out. PCA was then performed, followed by neighbor estimation and Leiden clustering.

An additional quality control step included glycolysis signature calculation. We followed a previously described approach^22^ by selecting genes that belong to the Gene Ontology term *‘*canonical_glycolysis’, using the tl.score_genes() function to estimate the glycolysis score. The same procedure was done for the term ‘aerobic electron transport chain’. Then, for each cell, we calculated differences between the glycolysis score and the aerobic score and filtered out Leiden clusters, which had median differences of more than 0.05. After that, highly variable genes were calculated again, followed by scaling and PCA as described above. Subsequently, we calculated new clusters using the Leiden algorithm and UMAP embeddings to obtain a 2D representation of the data.

To annotate the data, we first identified clusters using the RNA assay, based on the PCA embeddings, with the pp.neighbors(n_neighbors = 30, n_pcs = 60) and tl.leiden(default parameters) methods implemented in scanpy. Clusters were annotated into cell classes (NPC, neuroblast, neurons, neural crest derivatives) based on canonical marker expression. Based on neurotransmitter transporter expression, neuron clusters were further annotated into glutamatergic neurons, glycinergic neurons, motor neurons and dopaminergic neurons, and their subtypes were classified using canonical markers from the literature^86–91^.

To further decipher the heterogeneity of dopaminergic neurons, we subsetted neuroblasts and dopaminergic neuron clusters (21,901 cells) and repeated the reprocessing steps. To identify clusters, we used the pp.neighbors(n_neighbors = 30, n_pcs = 60) and tl.leiden(resolution = 0.3) methods implemented in scanpy. Clusters were annotated into dopaminergic neuron subtypes based on the canonical markers from the literature^64,66,92^. As in the time-course atlas, we did not observe *CALB1*^+^ or *ALDH1A1*^+^ cells, but identified *SOX6*^+^*, OTX2*^+^ and *Vglut2*^+^ subclusters, as well as an A9-like subtype expressing a high level of *KCNJ6*.

#### Determination of composition changes in the morphogen perturbation screen

To quantify differential cell type abundance under morphogen treatment compared to control, we used the speckle package^93^, which takes into account technical replicas of conditions. We used the propeller() function, using logit proportion transformation parameters. For each cell type, this function fits a linear model and estimates *P* values using two-sided *t*-tests, subsequently performing false discovery rate adjustment using the Benjamini–Hochberg procedure. For the estimation of differential dopaminergic cell abundance across conditions, we considered only conditions that had more than 200 dopaminergic neurons and followed the same steps as described above.

#### Estimation of AP and DV scores

To train the models to estimate AP and DV scores, the radial glia subset of a recently published first-trimester developing human brain transcriptomic atlas^28^ was used. In brief, the primary atlas was re-processed with scANVI^94^ with scVI^95^ model initialization plus cell type labels as the concatenation of the dissection region and cell class. Details have been described previously^22^.

Next, radial glial cells were selected for building the models. For the AP axis scoring model, the dissection tissues of the primary radial glial cells were first summarized to telencephalon, diencephalon, mesencephalon and rhombencephalon. Cells with a dissection tissue label that was too broad (for example, head and brain) were assigned to ‘none’. To correct for potential label errors resulting from mis-dissection, a label smoothening procedure was applied by calculating the number of neighboring cells dissected from each of the four summarized regions. A weighted proportion (using the Jaccard index for neighborhood similarity) was calculated, and the cell was reassigned to the region with the highest score. Based on the calibrated regional label, a value of 1, 2, 3 or 4 was assigned to each cell: 1 for telencephalon, 2 for diencephalon, 3 for mesencephalon and 4 for rhombencephalon. A total of 5,000 radial glial cells with each of the four values were then subset to train an elastic net model (with the glmnet package, default parameters) with the scANVI latent representation as the input. The AP score was validated using immunofluorescence microscopy through staining and imaging for HOXB4.

For the DV axis scoring model, the intuitive dorsal and ventral scores were first calculated as the module activity scores of the dorsal organizer markers (*LMX1A*, *DMRT3*, *MSX1*, *BNC2*, *MSX2*, *EYA4*, *MAFB*, *RXRG*, *OLIG3*) and ventral organizer markers (*TOX2, NKX2-2, FOXA2, NKX6-1, FOXJ1, LMX1B*, *FOXA1*, *PHOX2B*, *SIM1*, *POU3F1*, *NKX2-1*, *SIX6*, *SIX3*, *RAX*, *MAF*) using the AddModuleScores function in Seurat. Both marker sets were derived from the mouse developing brain atlas^96^. The marker-based DV scores were calculated as the difference between the two module scores (dorsal − ventral). A random subset of 80% of the radial glial cells with absolute values of the marker-based DV scores of >5 was selected to train an elastic net model (with the glmnet package, binomial family) to classify dorsal and ventral cells with the scANVI latent representation as the input.

To apply the two-scoring model to the organoid data, scArches^68^ was used to project the organoid data to the scANVI model of the primary atlas to obtain the projected latent representation. Next, the projected representation was used as the input to the trained AP and DV axis scoring model for prediction (type = ‘link’).

#### Mapping to primary data and estimation of gained populations

To map morphogen screen data to the primary human developing brain atlas, we used the approach described previously^22^ and mentioned above. In brief, scArches was used to map scRNA-seq data to the scANVI model of the human developing brain atlas. The sublibrary variable was chosen as a batch covariate. We trained the model for 100 epochs with weight_decay = 0 and the remaining default parameters. After mapping, we calculated the maximum presence scores of each condition for primary cells.

#### Trajectory inference based on cluster co-occurrence

We obtained the cell type proportions for different morphogen treatment conditions and calculated the pairwise Pearson correlation between cell types based on their proportions in different conditions. Using 1 − *r* as the distance, a cell type dendrogram and hierarchical clustering with the complete linkage function were applied to cell types annotated in the morphogen screening data. The number of clusters (*k* = 9) was determined manually to separate as many different NPC populations as possible and avoid neuron-only clusters. The resulting clusters of cell types were annotated based on the regional identities of the involved cell types.

#### Morpho-GRN inference with SCENIC

To infer the regulatory mechanisms of how different morphogens regulate changes in neural cell fate decisions, we adapted the morphogen-TF-regulon regulatory network inference approach described previously^36^. In brief, we first applied SCENIC^97^ to the morphogen screening scRNA-seq data to infer TF–target relationships. We generated 20 random subsets of the data, in each of which 400 cells per cell cluster (excluding cells in the neural crest and melanocyte clusters) were randomly selected. GRNBoost2 (ref. ^98^) was then applied to each of the subsets with the arboreto_with_multiprocessing.py code in pySCENIC (all with a given seed of ten). The TF–target pairs that appeared in at least three GRNBoost2 outputs were kept, with the maximum weights among all the estimated weights being used as the summary. The motif database v9 from cisTarget was then loaded to perform trimming based on TFBS motif enrichment based and correlation, given the summarized GRNBoost2 results and each of the 20 random subsets. Only TF–target pairs appearing at least three times among the 20 trimmings were retained as the final results. For each TF, the union of its predicted targets is considered as its regulon, and regulons with fewer than five genes were discarded. Next, the regulon activities were calculated with the tl.score_genes() function in the scanpy package in Python.

To then infer the regulatory relationship between morphogens and TF regulons, we ran GRNBoost2 with default parameters and seed = 10 using the arboreto_with_multiprocessing.py code in pySCENIC, given morphogen treatments (concentrations as values) as putative regulators and TF regulons (regulon module scores as values) as the putative targets. This analysis was only applied to the NPCs of the data, assuming that morphogen effects were on the commitment of different NPC populations.

Given that morphogen usages are unbalanced across conditions, we designed a subsampling strategy to target different numbers of cells for different conditions (Extended Data Fig. 9). First, we performed a hierarchical clustering of conditions based on their cell type enrichment relative to the control conditions, generating eight condition clusters. For each, conditions were further grouped into morphogen usage groups based on shared morphogen combinations, regardless of concentration. Next, we defined a cap of 1,000 NPCs as the targeted NPC number for every condition cluster during subsampling. This targeted number of NPCs per condition cluster was evenly assigned to its morphogen usage groups. Lastly, the targeted number of NPCs per morphogen usage group was again evenly assigned to the conditions, generating the target NPC number for each condition. During each subsampling, if a condition included more NPCs than the targeted number, the target number of NPCs was randomly selected; otherwise, all NPCs of the condition were always included. This random subsampling procedure was repeated 50 times to generate 50 NPC subsets.

Given each of the NPC subsets, GRNBoost2 was applied to estimate the regulatory strengths of morphogens on each TF regulon. For each morphogen–regulon pair, the estimated importances with the 50 different NPC subsets were summarized using the maximum. A threshold of estimated importance of >100 was used to screen for confident morphogen–regulon relationships. In addition, Pearson’s correlation was estimated between each morphogen usage concentration and regulon activities across all NPCs, and any morphogen–regulon pair with *r* < 0.1 was discarded.

#### Trajectory inference based on transcriptome similarity

Based on the mapping of the morphogen screening scRNA-seq dataset to the primary human developing brain atlas, as described above, we calculated the max–min normalized presence scores of each cell type annotated in the screening data for all cells in the primary atlas, following the approach outlined above. For each cell in the primary atlas, the maximum presence score across all cell types in the screening data (cell-type max presence scores) was calculated. Two criteria were used to identify primary cells matched with the screening cell types: cell-type max presence scores of >0.9, and cells in the primary cell cluster in which more than 80% of cells show cell-type max presence scores of >0.5. Any primary cell satisfying at least one of the two criteria was considered a matched primary cell and was included for the primary trajectory analysis (the matched primary sub-atlas).

Next, we subsetted the matched primary sub-atlas and re-identified 5,000 highly variable genes using the pp.highly_variable_genes() function in scanpy (batch_key = ‘Donor’). PCA was applied to the scaled expression of the identified highly variable genes, followed by Harmony integration^99^ to align the top 20 PCs of different donors. The UMAP embeddings for visualization of the primary sub-atlas were generated based on the harmony integration result.

To identify the matched population to the ventral medulla trajectory in the screening data, two cell-type max presence scores were calculated for each cell in the matched primary sub-atlas: the max presence scores for the ventral medulla cell types (*s*_vm_) including glutamatergic neuron (RELN high, glycinergic neuron (PAX2 high), glycinergic neuron (POU6F2 high, neuroblast medulla and NPC V-medulla; and the max presence scores of other cell types (*s*_o_). Cells with *s*_vm_ − *s*_o_ > 0.1 were considered the ventral medulla matched primary population.

To compare the matched ventral medulla trajectory in the two systems, we calculated NPC and neuron module scores with the AddModuleScore() function in Seurat for the screening data and the matched primary sub-atlas, given the NPC and neuron markers as described above. Next, we focused on the ventral medulla trajectory in the screening data and the matched ventral medulla trajectory in the primary sub-atlas. Genes were tested in each of the two datasets for their expression in relation to the neuron–NPC scores representing neural differentiation progress. This was done by using an *F*-test-based approach as described in a previous study^53^ to compare the residuals of two linear models: the full model including natural splines on the neuron–NPC scores (df = 5) and the reduced model with only the intercept. Genes with a Benjemini–Hochberg corrected *P* value of <0.01 were considered as differentiation-related differentially expressed. For the union of genes with differentiation-related differential expression in the two systems, we used a cubic smoothing spline model, implemented by the smooth.spline() function in R, to evenly interpolate expression along the trajectory (with neuron–NPC scores between −0.5 and 1.2). For each gene, Pearson’s correlation was calculated between the interpolated expression in the screening data and the primary data.

To validate the trajectories identified in the morphogen screening data based on cluster co-occurrence, we defined the confidently matched primary neuron populations to different neuron cell types identified in the morphogen screening dataset. In brief, we focused on primary cells with the neuron–NPC score of >0.75, and assigned a cell to a neuron cell type if its normalized presence score was at least 0.5 higher than the normalized presence score of any other neuron cell types. Similarly, we defined the confidently matched primary NPC populations to different NPC cell types identified in the morphogen screening data, focusing on primary cells with neuron–NPC scores of <0.

With both the neurons and NPCs labeled in the matched primary sub-atlas, we performed two different trajectory inference analyses on the matched primary sub-atlas. The first approach used CellRank2 with a hybrid of pseudotime kernel (with neuron–NPC scores as pseudotime, 50%) and connectivity kernel (with the unintegrated PCA, 50%), given neurons confidently matched to different neuron cell types as terminal states. The estimated likelihoods of different neuron cell types were summarized for NPCs confidently matched with different NPC populations in the screening data. The second approach was a stepwise neuron cell type label prediction along the differentiation trajectory, principally similar to FateID^100^. In brief, we binned cells in the matched primary sub-atlas into four groups based on their neuron–NPC scores: (−∞, −0.5], (−0.5, 0.25], (0.25, 0.75], (0.75, ∞). Starting from the last group, we trained an elastic net model with a multinomial family using the glmnet package in R, giving the assigned matched neuron cell type labels as the response variable and the Harmony-integrated PCs as the independent variables. The trained model was then applied to generate label predictions for the next group, and a new model was trained using cells in the next group, given the predicted label. This procedure was applied iteratively until the neuron cell type labels were assigned to all cells. Lastly, frequencies of predicted neuron cell type labels were counted for NPCs assigned to different NPC populations.

### Statistics and reproducibility

No statistical methods were used to pre-determine sample sizes, but our sample sizes are similar to those reported in previous publications^31^. Experiments were not randomized. Data collection and analysis were not performed blind to the conditions of the experiments. For the time-course and CRISPR-screen experiments, multiple organoids were pooled for better representation. For the morphogen screen, we have biological replicates. Exact numbers can be found in Supplementary Table 1. For the time-course experiment, we profiled three distinct iPS cell lines, and the results were consistent. For CRISPR-screen and morphogen perturbation experiments, we report no experimental covariates. For all box plots, the center line indicates the median, the box limits show the lower and upper quartiles and the whiskers extend to 1.5 times the interquartile range from the quartiles. Data met the assumptions of the statistical tests used. Immunohistochemistry analysis (Fig. 1b,c, Extended Data Fig. 1g–i and Extended Data Fig. 9b–e) was performed on two to three organoids per condition from one batch. The spatial transcriptomics experiment was performed on two organoids from one batch (Extended Data Fig. 3). For Fig. 4i, each boxplot corresponds to the clusters of the primary human brain atlas, and the number of samples equals the number of cells in the atlas^28^.

### Reporting summary

Further information on research design is available in the Nature Portfolio Reporting Summary linked to this article.

## Online content

Any methods, additional references, Nature Portfolio reporting summaries, source data, extended data, supplementary information, acknowledgements, peer review information; details of author contributions and competing interests; and statements of data and code availability are available at 10.1038/s41593-026-02316-x.

## Supplementary information

Reporting Summary

Supplementary Table 1 Overview of single-cell genomic experiments.

Supplementary Table 2 Cluster-specific markers of the posterior brain organoid atlas.

Supplementary Table 3 Differential expression between primary and organoid-derived dopaminergic neurons. *P* values were derived using ANOVA and FDR correction.

Supplementary Table 4 Genomic peak characterisation.

Supplementary Table 5 Peak cluster characterisation, including number of regions per cluster, top ten enriched GO-terms from GREAT analysis, and top ten enriched motifs.

Supplementary Table 6 Overview of gRNA sequences, primers for gRNA vector generation, and pooling strategy for KO experiments.

Supplementary Table 7 Transcriptomic KO effects in the CRISPR-perturbation screens for *EBF3*-KO, day 30, and *ONCECUT2*-KO, day 70. *P* values were derived using the Wilcoxon test and FDR correction.

Supplementary Table 8 Experimental conditions for probing of morphogen combinations in human neural posterior organoids. Dose and treatment windows of each morphogen are listed in separate columns, with culture days indicated at the top. The number of assayed organoids per morphogen treatment is indicated in the last column.

Supplementary Table 9 Enrichment effects in the morphogen screen. *P* values were derived using a *t*-test with FDR correction.

Supplementary Table 10 Gene panel used in MERSCOPE experiments.

## Data Availability

The count matrices and metadata for the RNA-seq portion of the time-course single-cell multi-omic dataset are part of the previously published integrated human neural organoid cell atlas, available on Zenodo (10.5281/zenodo.11203684) and the CellxGene Discover Census (https://cellxgene.cziscience.com/collections/de379e5f-52d0-498c-9801-0f850823c847). The raw and processed data of the scATAC-seq portion of the single-cell multi-omic data and the scRNA-seq data are deposited at Array Express with the following accession numbers: E-MTAB-15660, E-MTAB-15826 and E-MTAB-15659.

## Code availability

All code generated in the study, including analysis parameters, is available at https://github.com/quadbio/posterior_multiome. The Python package ATAC-mapper for mapping scATAC data of organoids to primary atlases is available at GitHub https://github.com/quadbio/atac_mapper and PyPi https://pypi.org/project/atac-mapper/.
