# Supplementary material for: Single-cell multi-omic atlas and morphogen screening informs midbrain and hindbrain organoid engineering
Source: Nat Neurosci. 2026 Jun 3;29(7):1548–58. doi: 10.1038/s41593-026-02316-x (PMC13337493; doi:10.1038/s41593-026-02316-x)
Supplement: Supplementary file 1 — Reporting Summary [file 41593_2026_2316_MOESM1_ESM.pdf]

nature portfolio

Corresponding author(s): J. Gray Camp
Barbara Treutlein

Last updated by author(s): 13.03.2026

# Reporting Summary

Nature Portfolio wishes to improve the reproducibility of the work that we publish. This form provides structure for consistency and transparency in reporting. For further information on Nature Portfolio policies, see our Editorial Policies and the Editorial Policy Checklist.

## Statistics

For all statistical analyses, confirm that the following items are present in the figure legend, table legend, main text, or Methods section.

| n/a | Confirmed | |
|---|---|---|
| ☐ | ☒ | The exact sample size (*n*) for each experimental group/condition, given as a discrete number and unit of measurement |
| ☐ | ☒ | A statement on whether measurements were taken from distinct samples or whether the same sample was measured repeatedly |
| ☐ | ☒ | The statistical test(s) used AND whether they are one- or two-sided<br>*Only common tests should be described solely by name; describe more complex techniques in the Methods section.* |
| ☐ | ☒ | A description of all covariates tested |
| ☐ | ☒ | A description of any assumptions or corrections, such as tests of normality and adjustment for multiple comparisons |
| ☐ | ☒ | A full description of the statistical parameters including central tendency (e.g. means) or other basic estimates (e.g. regression coefficient) AND variation (e.g. standard deviation) or associated estimates of uncertainty (e.g. confidence intervals) |
| ☐ | ☒ | For null hypothesis testing, the test statistic (e.g. *F*, *t*, *r*) with confidence intervals, effect sizes, degrees of freedom and *P* value noted<br>*Give P values as exact values whenever suitable.* |
| ☒ | ☐ | For Bayesian analysis, information on the choice of priors and Markov chain Monte Carlo settings |
| ☒ | ☐ | For hierarchical and complex designs, identification of the appropriate level for tests and full reporting of outcomes |
| ☐ | ☒ | Estimates of effect sizes (e.g. Cohen's *d*, Pearson's *r*), indicating how they were calculated |

*Our web collection on statistics for biologists contains articles on many of the points above.*

## Software and code

Policy information about availability of computer code

| | |
|---|---|
| Data collection | Please, refer to Experimental methods section |
| Data analysis | Please, refer to Data analysis methods section |

For manuscripts utilizing custom algorithms or software that are central to the research but not yet described in published literature, software must be made available to editors and reviewers. We strongly encourage code deposition in a community repository (e.g. GitHub). See the Nature Portfolio guidelines for submitting code & software for further information.

## Data

Policy information about availability of data

All manuscripts must include a data availability statement. This statement should provide the following information, where applicable:

- Accession codes, unique identifiers, or web links for publicly available datasets
- A description of any restrictions on data availability
- For clinical datasets or third party data, please ensure that the statement adheres to our policy

The RNA portion of time course data is a part of published integrated human neural organoid cell atlas which is available at zenodo (https://doi.org/10.5281/zenodo.11203684) and the CellxGene Discover Census (https://cellxgene.cziscience.com/collections/de379e5f-52d0-498c-9801-0f850823c847). All the raw data is uploaded to Array Express with the following accession numbers: E-MTAB-15660, E-MTAB-15826, E-MTAB-15659.

## Research involving human participants, their data, or biological material

Policy information about studies with human participants or human data. See also policy information about sex, gender (identity/presentation), and sexual orientation and race, ethnicity and racism.

| | |
|---|---|
| Reporting on sex and gender | NA |
| Reporting on race, ethnicity, or other socially relevant groupings | NA |
| Population characteristics | NA |
| Recruitment | NA |
| Ethics oversight | NA |

Note that full information on the approval of the study protocol must also be provided in the manuscript.

# Field-specific reporting

Please select the one below that is the best fit for your research. If you are not sure, read the appropriate sections before making your selection.

☒ Life sciences ☐ Behavioural & social sciences ☐ Ecological, evolutionary & environmental sciences

For a reference copy of the document with all sections, see nature.com/documents/nr-reporting-summary-flat.pdf

# Life sciences study design

All studies must disclose on these points even when the disclosure is negative.

| | |
|---|---|
| Sample size | For determining the number of cells to be sequenced per organoid, the analysis done in our project is as extensive as any previous study; based on Kanton et al. (Nature 2019), Fleck et al. (Nature 2023), Zenk et al. (Nature Neuroscience 2024), we believe that we sufficiently sample the heterogeneity with the number of cells sequenced. See Supplementary Table 1. |
| Data exclusions | We excluded low quality cells using criteria as described in the Methods |
| Replication | We analyzed multiple organoids from multiple cell lines for each different time point in the time course and multiple organoids for CRISPR and morphogen perturbation experiment. |
| Randomization | Experiments were not randomized. |
| Blinding | Investigators were not blinded during data acquisition. |

# Reporting for specific materials, systems and methods

We require information from authors about some types of materials, experimental systems and methods used in many studies. Here, indicate whether each material, system or method listed is relevant to your study. If you are not sure if a list item applies to your research, read the appropriate section before selecting a response.

## Materials & experimental systems

| n/a | Involved in the study | |
|---|---|---|
| ☐ | ☒ | Antibodies |
| ☐ | ☒ | Eukaryotic cell lines |
| ☒ | ☐ | Palaeontology and archaeology |
| ☒ | ☐ | Animals and other organisms |
| ☒ | ☐ | Clinical data |
| ☒ | ☐ | Dual use research of concern |
| ☒ | ☐ | Plants |

## Methods

| n/a | Involved in the study | |
|---|---|---|
| ☒ | ☐ | ChIP-seq |
| ☐ | ☒ | Flow cytometry |
| ☒ | ☐ | MRI-based neuroimaging |

## Antibodies

| | |
|---|---|
| Antibodies used | rabbit anti-MAP2 (1:1000, Sigma-Aldrich, AB5622), mouse anti-OTX2 (1:200, Invitrogen, MA5-15854), goat anti-FOXA2 (1:200, R&D systems, AF2400), mouse anti-TH (1:70, Novus Biologicals, MAB7566), chicken anti-CALB1 (1:1000, Novus Biologicals, |

| | |
|---|---|
| | NBP2-50028SS), guinea pig anti-VGLUT1 (1:250, Merck, AB5905), rabbit anti-LHX9 (1:30, Merck, HPA009695), chicken anti-Doublecortin (1:1000, Abcam, ab153668), mouse anti-PAX2 (1:500, Antibodies.com, A252739), goat anti-ZIC1 (1:40, Thermo Fisher Scientific, PA5-47681), rabbit anti-GlyT2 (1:500, Thermo Fisher Scientific, MA5-52662). 1:300 secondary antibody (Donkey: anti-rabbit AF 488, Invitrogen, A32790; anti-rabbit AF 568, Invitrogen, A10042; anti-rabbit AF 647, Invitrogen, A31573; anti-mouse AF 568, Invitrogen, A10037; anti-chicken AF 488, Invitrogen, A78948; anti-goat AF 488, Invitrogen, A11055; anti-goat AF 647, Invitrogen, A21447; anti-guinea pig AF 488, Jackson Immunoresearch (Lucerna Chem,) 706-545-148. Goat: anti-rabbit AF 488, Invitrogen, A11034; anti-mouse AF 568, Invitrogen, A11031; anti-chicken AF 647, Invitrogen, A32933; anti-guinea pig AF 568, Invitrogen, A11075). |
| Validation | Validations as provided by manufacturer. |

# Eukaryotic cell lines

Policy information about cell lines and Sex and Gender in Research

| | |
|---|---|
| Cell line source(s) | See methods section "Stem cell and organoid culture" . |
| Authentication | See methods section "Stem cell and organoid culture". |
| Mycoplasma contamination | All cell lines and organoids, grown from cell lines were regularly tested for mycoplasma using a PCR-based test and were found to be negative for mycoplasma. |
| Commonly misidentified lines (See ICLAC register) | None. |

# Plants

| | |
|---|---|
| Seed stocks | NA |
| Novel plant genotypes | NA |
| Authentication | NA |

# Flow Cytometry

## Plots

Confirm that:

☒ The axis labels state the marker and fluorochrome used (e.g. CD4-FITC).

☒ The axis scales are clearly visible. Include numbers along axes only for bottom left plot of group (a 'group' is an analysis of identical markers).

☐ All plots are contour plots with outliers or pseudocolor plots.

☐ A numerical value for number of cells or percentage (with statistics) is provided.

## Methodology

| | |
|---|---|
| Sample preparation | Please refer to methods section "Stable cell line and mosaic organoids generation for perturbation experiment". |
| Instrument | BD FACS Aria Fusion and FACSAria III |
| Software | FlowJo |
| Cell population abundance | Please refer to Extended Data Figure 6 |
| Gating strategy | See Extended Data Figure 6. Three gates were used 1) FSC-A vs SSC were used to gate for the bulk population of cells, 2) FCS-A vs FSC-H was used to minimize doublet sorting, 3) GFP+ and RFP+ populations were determined and gated by comparing to respective negative controls. |

☒ Tick this box to confirm that a figure exemplifying the gating strategy is provided in the Supplementary Information.
